# Scope and Limitations of 3‐Iodo‐Kdo Fluoride‐Based Glycosylation Chemistry using *N*‐Acetyl Glucosamine Acceptors†

**DOI:** 10.1002/open.201500126

**Published:** 2015-07-29

**Authors:** Barbara Pokorny, Paul Kosma

**Affiliations:** ^1^ Department of ChemistryUniversity of Natural Resources and Life Sciences-ViennaMuthgasse 181190ViennaAustria

**Keywords:** glycosylation, hydrogen transfer, iodonium, Kdo, lipopolysaccharide

## Abstract

The ketosidic linkage of 3‐deoxy‐d‐*manno*‐octulosonic acid (Kdo) to lipid A constitutes a general structural feature of the bacterial lipopolysaccharide core. Glycosylation reactions of Kdo donors, however, are challenging due to the absence of a directing group at C‐3 and elimination reactions resulting in low yields and anomeric selectivities of the glycosides. While 3‐iodo‐Kdo fluoride donors showed excellent glycosyl donor properties for the assembly of Kdo oligomers, glycosylation of *N*‐acetyl‐glucosamine derivatives was not straightforward. Specifically, oxazoline formation of a β‐anomeric methyl glycoside, as well as iodonium ion transfer to an allylic aglycon was found. In addition, dehalogenation of the directing group by hydrogen atom transfer proved to be incompatible with free hydroxyl groups next to benzyl groups. In contrast, glycosylation of a suitably protected methyl 2‐acetamido‐2‐deoxy‐α‐d‐glucopyranoside derivative and subsequent deiodination proceeded in excellent yields and α‐specificity, and allowed for subsequent 4‐O‐phosphorylation. This way, the disaccharides α‐Kdo‐(2→6)‐α‐GlcNAcOMe and α‐Kdo‐(2→6)‐α‐GlcNAcOMe‐4‐phosphate were obtained in good overall yields.

## Introduction

The eight‐carbon sugar 3‐deoxy‐d‐*manno*‐oct‐2‐ulosonic acid (Kdo) is a biomedically important constituent of bacterial polysaccharides occurring in capsular polysaccharides (CPS), O‐antigens, and the core region of lipopolysaccharides (LPS). Whereas Kdo has been found in both anomeric configurations in CPS, the LPS of many Gram‐negative bacteria harbors a structurally conserved α‐(2→4)‐linked Kdo‐disaccharide which connects the endotoxically active lipid A part to the core region and the O‐antigenic polymer.^1^ Lipid A is composed of a bisphosphorylated β‐(1→6)‐linked N‐/O‐acylated glucosamine disaccharide which plays a decisive role in the immune response of host cells when infected by Gram‐negative bacteria.^2^ Kdo is present in an acid‐labile α‐ketosidic linkage to position 6 of the distal glucosamine unit. Whereas the acylated, bisphosphorylated diglucosamine unit provides the main interactions in the complex with Toll‐like receptor 4/myelodifferentiation factor MD‐2, the Kdo and adjacent heptose and outer core sugars provide additional binding epitopes for receptor interactions and recognition by antibodies and lectins.^3^

Thus, the chemical synthesis of relevant Kdo‐lipid A fragments constitutes an important target and has successfully been pursued by several groups.^4^ Coupling of Kdo donors, however, is far from trivial, and an elaborate optimization of protecting and anomeric leaving groups for each glycosylation step is often crucial for good results.^5^, ^6^ Inherent challenges in glycosidation reactions of 3‐deoxy‐2‐ulosonic acid glycosyl donors pertain to the absence of a stereodirecting group at C‐3, resulting in low anomeric selectivities. Furthermore, competing elimination reactions are common and are promoted by the deactivating C‐1 ester group. In our previous work we have presented a convenient approach towards the α‐specific and regioselective synthesis of Chlamydia‐related Kdo oligomers using 3‐iodo‐Kdo fluoride donor **1** for the coupling step followed by deiodination of the stereodirecting auxiliary group.^7^ Gratifyingly, the elimination reaction could also be largely suppressed, and the protocol was successfully expanded to include the formation of the sterically demanding α‐(2→5)‐linkage of Kdo units.^8^ In continuation of these applications, we have set out to investigate the suitability of donor **1** for the glycosylation of glucosamine acceptors, specifically addressing regioselective glycosylation at position 6 with the option for subsequent phosphorylation at position 4 in order to generate the common phosphoester substitution at the distal glucosamine unit of lipid A.^2^, ^9^

## Results and Discussion

**Scheme 1:**
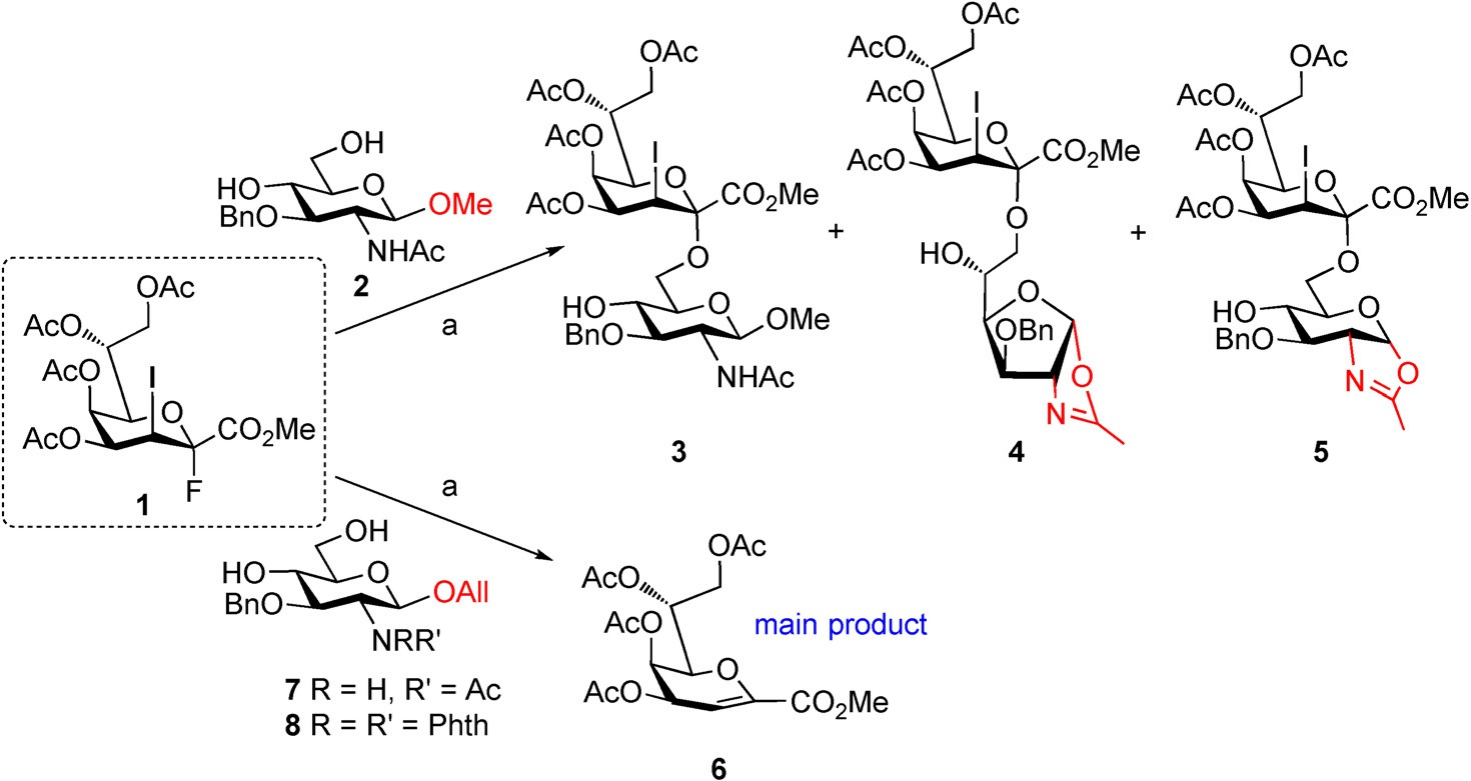
*Reagents and conditions*: a) BF_3_⋅Et_2_O, 3 Å molecular sieves, CH_2_Cl_2_, 0 °C→rt, 1.5 h, **3**: 32 %, **4**: 11 %, **5**: 3 %.

As glycosyl acceptors, *N*‐acetyl‐protected monosaccharide derivatives were selected, since glycosylation of the primary alcohol of *N*‐acetyl‐, *N*‐acyl‐, *N*‐Cbz‐, and *N*‐Troc‐protected glucosamine derivatives with various Kdo donors gave comparable yields and anomeric selectivities.^10^ In a first trial experiment, the easily accessible *N*‐acetyl‐β‐d‐glucosamine methyl glycoside derivative **2** 11 was coupled with the previously described peracetylated 3‐iodo‐Kdo fluoride donor **1** 7 under BF_3_^.^Et_2_O‐promotion in dichloromethane (Scheme 1). Provided, that a regioselective reaction at position 6 would be achieved, the 4‐OH group should remain accessible for subsequent phosphate introduction, thus minimizing protecting group manipulations.

Disappointingly, disaccharide **3** was isolated in poor yield (32 %). As expected, only traces of elimination product **6** 12 were found and formation of the corresponding β‐linked Kdo‐GlcNAc disaccharide was not observed. As the main disaccharide side products, furanose **4** (11 %) and pyranose **5** oxazolines (3 %) were identified. The structure of the furanose ring form of **4** was proven by an heteronuclear multiple bond correlation (HMBC) between H‐1 and C‐4. When donor **1** was reacted with allyl glycoside **7** (see Supporting Information) under similar glycosylation conditions, a complex mixture of degradation products was obtained. Interestingly, a pronounced elimination of donor **1** giving glycal ester **6** was observed on thin‐layer chromatography (TLC).

**Scheme 2:**
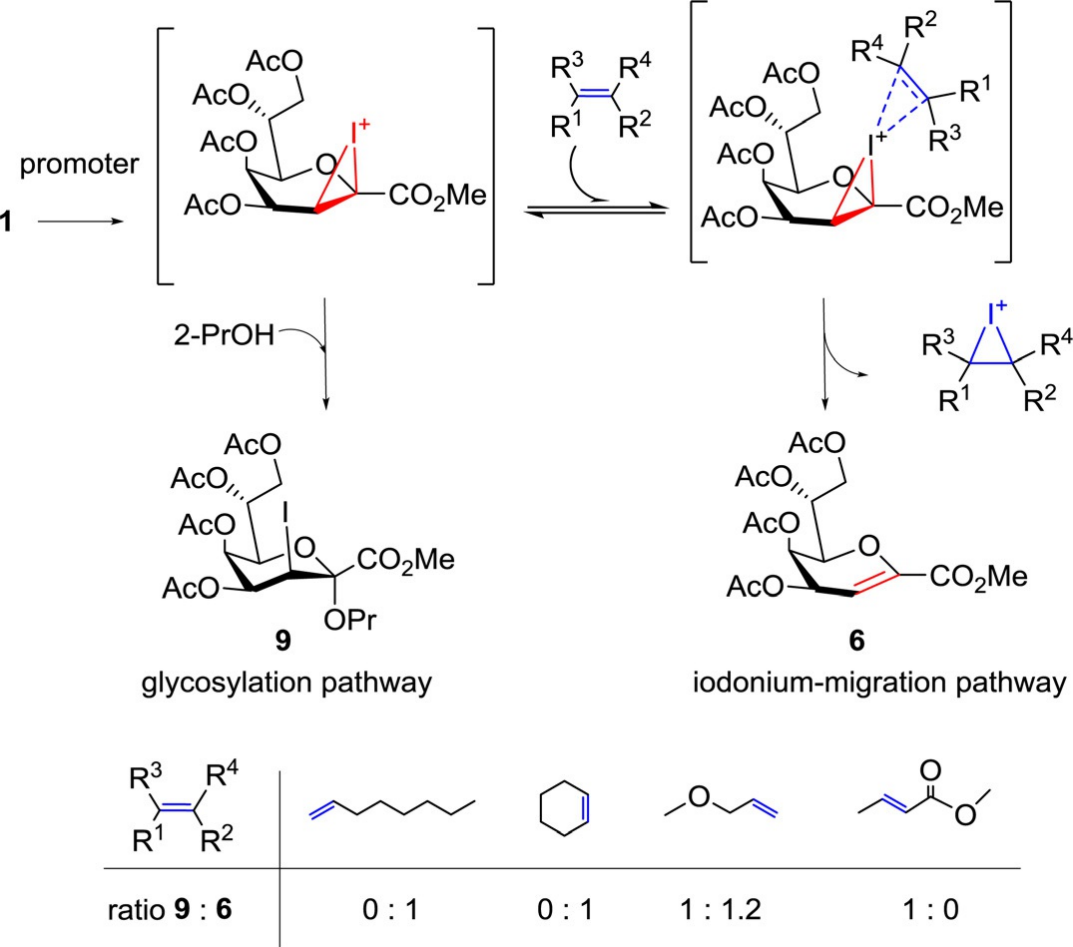
Crossover experiments of intermediary iodonium ion in the presence of different olefins and ratios of obtained product mixtures.

In a preliminary experiment, the same behavior was also noted for the *N*‐phthalimido‐protected β‐allyl glycosyl acceptor **8** (see Supporting Info). This outcome prompted us to investigate the reaction mechanism in more detail, since this high propensity of 3‐iodo fluoride donor **1** towards elimination had previously not been observed.^7^ We hypothesized that fast and irreversible migration of an intermediary iodonium ion^13^, ^14^ to the allylic double bond leads to formation of the glycal ester **6** and outcompetes glycoside formation (Scheme 2).

We expected that a transfer of iodonium ion generated from the activated donor to the allyl aglycon could be rationalized by the distinct difference in electron density of the allyl aglycon when compared to the rather electron‐deficient α,β‐unsaturated ester **6**. To investigate this hypothesis, the competitive behavior of different olefins in a coupling reaction of donor **1** with 2‐propanol in dichloromethane under BF_3_^.^Et_2_O‐promotion was screened (for experimental detail see Supporting Information). The reaction was performed and directly monitored in an NMR tube. The consumption of the respective olefin was determined by comparing representative ^1^H NMR signals before and after donor activation in relation to the residual solvent peak. The decrease of olefin matched the amount of glycal **6** formed in the mixture. Immediate and intensive discoloration of the solutions upon activation indicated the instability of the resulting alkyl halide structures under the applied conditions that prevented identification of the iodo species by NMR. In fact the unsubstituted olefins 1‐octene and cyclohexene suppressed formation of the known 2‐propyl glycoside **9** 7 and donor **1** degraded completely to glycal **6** within a few seconds. Next we used allyl methyl ether resembling the aglycon of acceptors **7** and **8**. Parallel formation of glycoside **9** and glycal **6** (final composition of the mixture was based on relative ^1^H NMR integral values: **9**:**6**=1:1.2) over a period of 1 h indicated that both pathways (Scheme 2) are operative. In contrast, in the presence of α,β‐unsaturated methyl crotonate, glycoside **9** was obtained without any formation of elimination product **6**. The observation that this electrophilic double bond does not inhibit the glycosylation reaction was in agreement with our previous result that donor **1** readily glycosylates glycal ester acceptors.^7^ In summary, electronic rather than steric effects seem to dictate the halonium migration—a trend that is conclusively reflected by the different results obtained for the two terminal olefins 1‐octene and allyl methyl ether. This may further explain why 2‐iodo‐2‐deoxy sugar donors, that—in comparison to ulosonic acids like Kdo—lack a deactivating C‐1 ester group next to the anomeric center, are not affected by a competitive iodonium‐ion migration in the presence of allyl groups.^15^ Thus, the allyl group turned out to be an unsuitable protecting or aglyconic group for the 3‐iodo‐Kdo fluoride donor **1** under these conditions.

**Scheme 3:**
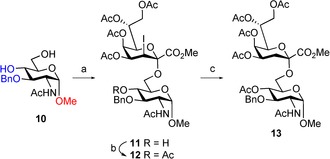
*Reagents and conditions*: a) **1**, BF_3_⋅Et_2_O, 3 Å molecular sieves, CH_2_Cl_2_, 0 °C→rt, 1 h, 55 %; b) Ac_2_O, 4‐(*N*,*N*‐dimethylamino)pyridine, pyridine, rt, 5 h, 59 %; c) lauroyl peroxide, cyclohexane/1,2‐dichloroethane (8:1), reflux, 2 h, 75 %.

Next, the α‐anomeric methyl glycoside **10** (see Supporting Information) was subjected to glycosylation (Scheme 3). In contrast to the outcome of the reaction with β‐anomeric glycoside **2**, the resulting disaccharide **11** was not susceptible to oxazoline formation under the applied conditions. The coupling reaction with sparingly soluble acceptor **10** provided disaccharide **11** in moderate yield (∼55 %) together with an inseparable impurity (∼5 % based on the ^1^H NMR data). First, we believed in the presence of the 4‐*O*‐regioisomer as only one *m/z* value was observed for the mixture. However, the expected low‐field‐shifted ^13^C NMR signal of C‐4 and high‐field‐shifted signal of C‐6, respectively, were not detected. Instead, signals of C‐3 and the benzylic carbon were shifted to lower field (see Supporting Information) in comparison to the major compound. For further analysis, **11** was submitted to the previously optimized dehalogenation procedure capitalizing on a hydrogen atom transfer reaction using lauroyl peroxide in cyclohexane (Scheme 3).^16^ Free hydroxyl groups were usually tolerated by this method in previous experiments, although decreased solubility of only partially protected substrates required prolonged reaction times.^7^ Notably, the free 4‐hydroxyl group vicinal to the benzyl group led to a significantly decreased yield. Among different side products, an acid labile 3,4‐*O*‐benzylidene protected disaccharide could be identified. Similar observations have been described by the Bols group, who later capitalized on this benzylidene formation and its ensuing cleavage as a regioselective deprotection method for benzyl groups vicinal to free OH groups.^17^ In agreement with their observation that free hydroxyl groups were essential for this reaction type17a—involving a radical process17b—benzyl groups did not interfere with the hydrogen atom transfer reaction when using fully protected carbohydrate moieties. Further on, the disaccharide **11** containing the unknown entity was O‐acetylated (**12**) prior to dehalogenation that provided disaccharide **13** in 75 % yield. Neither of these steps allowed separation of the minor unit by high‐performance liquid chromatography (HPLC). Again, the only significant differences were seen for the low‐field NMR shifts of C‐3 and the benzylic carbon of the minor species. Thus, we surmised the presence of two stable rotamers in the disaccharide product.

**Figure 1:**
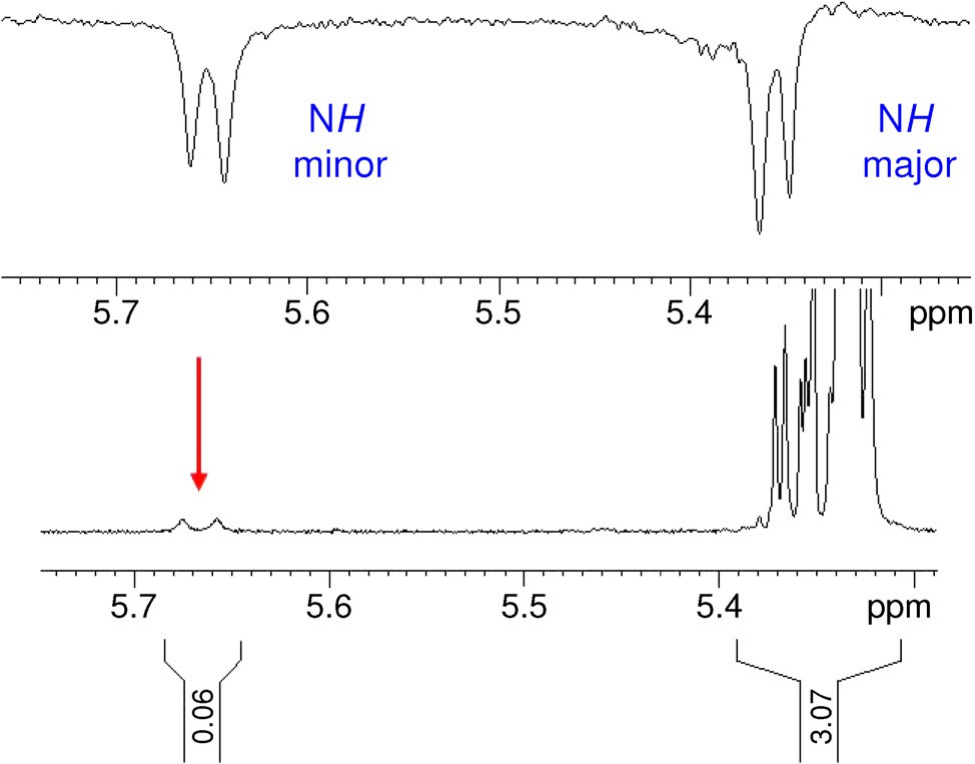
Evidence for the presence of two rotamers in disaccharide **13**; top: 1D NOE‐difference spectrum after selective pulse at 5.64 ppm (N*H* signal of minor compound); bottom: expansion plot of ^1^H NMR of **13** showing the NH signals.

This was eventually proven by a method proposed by Ley's group wherein 1 D‐nuclear Overhauser effect (NOE)‐difference spectra are used to distinguish between equilibrating rotamers and nonequilibrating isomers.^18^ By selective excitation of the N*H* signal of the minor entity, a parallel attenuation of the distant and heavily overlapped N*H* of the major species was observed (Figure 1). This phenomenon only occurs for the equilibrating rotamers but not for chemically distinct isomers.

**Scheme 4:**
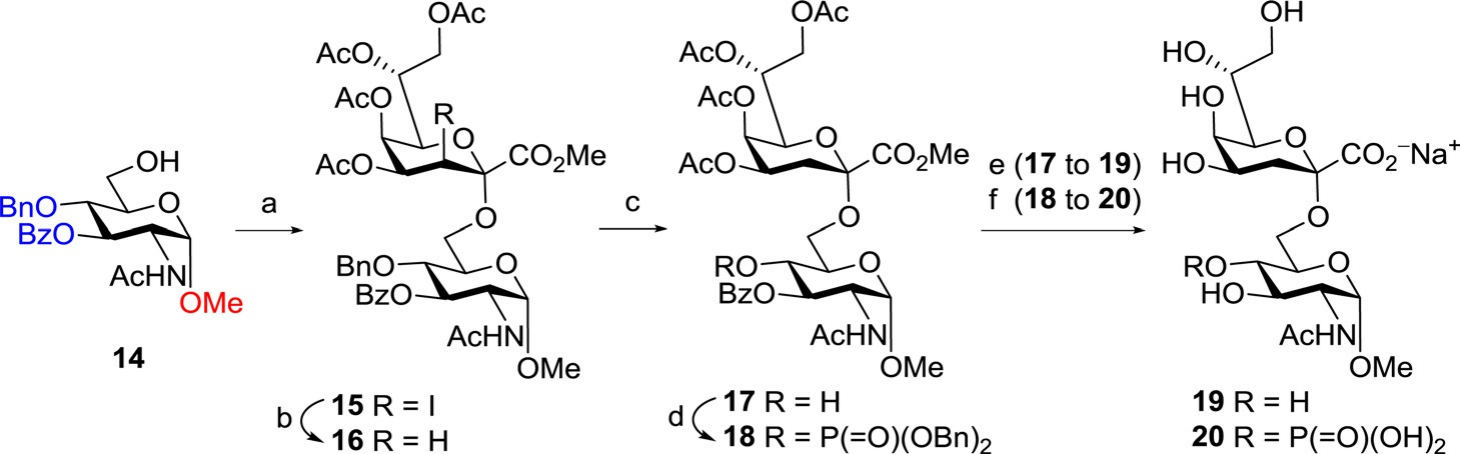
*Reagents and conditions*: a) **1**, BF_3_⋅Et_2_O, 3 Å molecular sieves, CH_2_Cl_2_, 0 °C→rt, 1 h, 89 %; b) lauroyl peroxide, cyclohexane/1,2‐dichloroethane (8:1), reflux, 2 h, 87 %; c) Pd/C (10 %), H_2_, MeOH, rt., 3 h, 96 %; d) dibenzyl *N*,*N*‐diisopropylphosphoramidite, 1*H*‐tetrazole, 4 Å molecular sieves, CH_2_Cl_2_, 0 °C, 70 min, then *m*CPBA, 0 °C, 1 h, 70 %; e) NaOMe, MeOH, 3 h, then aq. NaOH, 0 °C, 5 h, 97 %; f) Pd/C (10 %), H_2_, MeOH, rt, 30 min, then NaOMe, MeOH, rt, 17 h, then aq NaOH, 0 °C, 5 h, 99 %.

To increase the limited solubility of the acceptor and to avoid problems at the dehalogenation step, the protecting group pattern of the GlcNAc acceptor was revised and the 3‐*O*‐benzoyl‐4‐*O*‐benzyl‐protected acceptor **14** (see Supporting Information) was coupled with 3‐iodo fluoride donor **1** following the general protocol (Scheme 4). This way, disaccharide **15** was obtained in a high yield (89 %) as the α‐anomer only,^19^ and was dehalogenated (87 % of **16**) by hydrogen atom transfer.^20^ Debenzylation by catalytic hydrogenation (Pd/C, H_2_) afforded 4‐OH disaccharide **17** (96 %). Subsequent phosphorylation capitalizing on the two‐step procedure using dibenzyl *N,N*‐diisopropylphosphoramidite/1*H*‐tetrazole followed by oxidation with *meta*‐chloroperoxybenzoic acid (*m*CPBA) yielded 4‐*O*‐phosphotriester **18** (70 %).^21^

Compound **17** was subjected to transesterification with methanol under Zemplén conditions (the formed methyl benzoate had to be removed by repeated extraction between water and *n*‐hexane), followed by saponification of the methyl ester to give disaccharide **19** in 97 % yield. For global deprotection of **18**, the phosphotriester was first debenzylated affording the free phosphate, which was no longer prone to migration/hydrolysis under basic conditions. Deprotection of the acyl groups and ester hydrolysis furnished the deblocked disaccharide **20** as sodium salt in near theoretical yield.

## Conclusions

3‐Iodo‐Kdo fluoride donor **1** was suitable for stereo‐ and regioselective glycosylation of a 6‐OH GlcNAc acceptor, but high yields relied on an appropriate protecting group pattern. The acceptor had to be designed as the α‐OMe glycoside to avoid oxazoline formation. Ensuing dehalogenation by hydrogen atom transfer reaction did not tolerate free hydroxyl groups in close proximity to the benzyl protecting group. Furthermore, the 3‐iodo group was incompatible with the presence of nucleophilic olefins due to putative iodonium ion migration resulting in glycal ester **6**. Within these boundary conditions, however, the α‐specific glycosylation, dehalogenation, and global deprotection proceeded in high overall yields.

## Experimental Section

### Chemistry

**General**: All purchased chemicals were used without further purification unless stated otherwise. Solvents (CH_3_CN, CH_2_Cl_2_, cyclohexane, 1,2‐dichloroethane, *N,N*‐dimethylformamide) were dried over activated 4 Å molecular sieves. Dry MeOH (secco solv) was purchased from Merck. Cation exchange resin DOWEX 50 H^+^ was regenerated by consecutive washing with HCl (3 m), water, and dry MeOH. The promoter BF_3_⋅Et_2_O was used as a solution in diethyl ether (≥46 % according to the manufacturer). Concentration of organic solutions was performed under reduced pressure<40 °C. Optical rotations were measured with a PerkinElmer 243 B Polarimeter (Waltham, USA). [α]_D_^20^ values are given in units of 10^−1^ deg cm^2^ g^−1^. Thin layer chromatography was performed on Merck precoated plates: generally on 5×10 cm, layer thickness 0.25 mm, Silica Gel 60F_254_; alternatively on HP‐TLC plates with 2.5 cm concentration zone (Merck). Spots were detected by dipping reagent (anisaldehyde‐H_2_SO_4_). For column chromatography, silica gel (0.040–0.063 mm) was used. HP‐column chromatography was performed on a prepacked column (YMC‐Pack SIL‐06, 0.005 mm, 250×10 mm). Size exclusion chromatography for desalting was performed on prepacked PD‐10 columns (GE Healthcare, Sephadex G‐25 m). NMR spectra were recorded with a Bruker Avance III 600 instrument (600.22 MHz for ^1^H, 150.93 MHz for ^13^C and 242.97 MHz for ^31^P) using standard Bruker NMR software (Rheinstetten, Germany). ^1^H spectra were referenced to 7.26 (CDCl_3_) and 0.00 (D_2_O, external calibration to 2,2‐dimethyl‐2‐silapentane‐5‐sulfonic acid) ppm unless stated otherwise. ^13^C spectra were referenced to 77.00 (CDCl_3_) and 67.40 (D_2_O, external calibration to 1,4‐dioxane) ppm. ^31^P spectra in D_2_O were referenced to external *ortho*‐phosphoric acid (0.00 ppm). Electrospray ionization mass spectrometry (ESI‐MS) data were obtained on a Waters Micromass Q‐TOF Ultima Global instrument (Santa Clara, USA) or on a Thermo Scientific Exactive Plus Orbitrap instrument (Waltham, USA).

**Methyl (4,5,7,8‐tetra‐*O*‐acetyl‐3‐deoxy‐3‐iodo‐d‐*glycero*‐α‐d‐*talo*‐oct‐2‐ulopyranosyl)onate‐(2→6)‐methyl 2‐acetamido‐3‐*O*‐benzoyl‐4‐*O*‐benzyl‐2‐deoxy‐α‐d‐glucopyranoside (15)**: A suspension of 3‐iodo‐Kdo donor **1** (76.6 mg, 0.140 mmol) and acceptor **14** (50.0 mg, 0.116 mmol) in dry CH_2_Cl_2_ (4.0 mL) containing ground 3 Å molecular sieves (200 mg) was stirred for 1 h at ambient temperature. The cooled mixture (0 °C) was treated with BF_3_⋅Et_2_O (44 μL, 0.349 mmol) and kept at ambient temperature for 1 h. After dilution with CH_2_Cl_2_, the organic phase was washed successively with satd. NaHCO_3_ (1×5 mL), sodium thiosulfate (5 *w* %) (1×5 mL), and brine (1×5 mL). The solution was dried over MgSO_4_, filtered, and concentrated, and the crude product was purified by chromatography (toluene/EtOAc 1:2) affording disaccharide **15** as a colorless oil (99.2 mg, 89 %): *R*_f_=0.31 (toluene/EtOAc 1:2, HP‐TLC); [α]_D_^20^=+68.2 (*c*=1.28 in CHCl_3_); ^1^H NMR (CDCl_3_): *δ*=8.03–8.00 (m, 2 H, Ar), 7.58–7.55 (m, 1 H, Ar), 7.46–7.42 (m, 2 H, Ar), 7.25–7.08 (m, 5 H, Ar), 5.82 (d, 1 H, *J*_N*H*,2_ 9.5 Hz, N*H*), 5.55 (dd, 1 H, *J*_3,2_ 10.8, *J*_3,4_ 9.1 Hz, H‐3), 5.38–5.36 (m, 1 H, H‐5′), 5.34 (ddd, 1 H, *J*_7′,6’_ 9.5, *J*_7′,8′b_ 4.1, *J*_7′,8′a_ 2.6 Hz, H‐7′), 5.05 (dd, 1 H, *J*_4′,3’_ 4.7, *J*_4′,5’_ 3.7 Hz, H‐4′), 4.72 (d, 1 H, *J*_1,2_ 3.5 Hz, H‐1), 4.67 (dd, 1 H, *J*_8′a,8′b_ 12.3 Hz, H‐8′a), 4.64 (d, 1 H, *J* 11.2 Hz, C*H*HPh), 4.51 (d, 1 H, H‐3′), 4.44 (d, 1 H, *J* 11.2 Hz, CH*H*Ph), 4.36 (ddd, 1 H, H‐2), 4.32 (dd, 1 H, *J*_6′,5’_ 1.8 Hz, H‐6′), 4.16 (dd, 1 H, H‐8′b), 3.93–3.89 (m, 1 H, H‐5), 3.74 (s, 3 H, CO_2_C*H_3_*), 3.61–3.52 (m, 3 H, H‐4, H‐6a, H‐6b), 3.42 (s, 3 H, OC*H_3_*), 2.11, 2.05, 2.03, 1.99, and 1.83 ppm (5 s, each 3 H, COC*H_3_*); ^13^C NMR (CDCl_3_): *δ*=170.3, 170.1, 170.0, 169.5, and 169.3 (5 s, 5C, *C*OCH_3_), 166.8 (s, 1C, *C*OPh), 165.6 (s, C‐1′), 137.0 (s, 1C, Ar), 133.4 (d, 1C, Ar), 129.7 (d, 2C, Ar), 129.3 (s, 1C, Ar), 128.5 (d, 2C, Ar), 128.4 (d, 2C, Ar), 128.0 (d, 1C, Ar), 127.9 (d, 2C, Ar), 101.4 (s, C‐2′), 98.0 (d, C‐1), 76.3 (d, C‐4), 74.9 (t, *C*H_2_Ph), 74.2 (d, C‐3), 69.8 (d, C‐5), 68.3 (d, C‐6′), 67.7 (d, C‐7′), 65.2 (d, C‐4′), 65.1 (t, C‐6), 63.4 (d, C‐5′), 61.8 (t, C‐8′), 55.1 (q, O*C*H_3_), 52.8 (q, CO_2_ *C*H_3_), 52.3 (d, C‐2), 23.1 (q, CO*C*H_3_), 21.8, 20.9, 20.8, 20.7, and 20.6 ppm (4 q, 1 d, 5C, 4×CO*C*H_3_, C‐3′); HRMS (ESI‐TOF) *m/z* [*M*+Na]^+^ calcd for C_40_H_48_INO_18_Na^+^: 980.1808, found: 980.1808.

**Methyl (4,5,7,8‐tetra‐*O*‐acetyl‐3‐deoxy‐α‐d‐*manno*‐oct‐2‐ulopyranos‐yl)onate‐(2→6)‐methyl 2‐acetamido‐3‐*O*‐benzoyl‐4‐*O*‐benzyl‐2‐deoxy‐α‐d‐glucopyranoside (16)**: Disaccharide **15** (99.0 mg, 0.103 mmol) was dissolved in dry cyclohexane (4.0 mL) and dry 1,2‐dichloroethane (0.5 mL) and the solution was degassed with argon. After heating to reflux for 15 min, lauroyl peroxide (14.4 mg, 0.036 mmol) was added and the solution was held at reflux for 2 h. The mixture was concentrated in vacuo, and the residue was subjected to chromatography (*n*‐hexane/EtOAc 1:9), yielding dehalogenated compound **16** as a colorless oil (75.0 mg, 87 %); *R*_f_=0.36 (CH_2_Cl_2_/EtOAc 1:1, HP‐TLC); [α]_D_^20^=+82.2 (*c*=0.66 in CHCl_3_); ^1^H NMR (CDCl_3_): *δ*=8.02–8.00 (m, 2 H, Ar), 7.58–7.54 (m, 1 H, Ar), 7.46–7.42 (m, 2 H, Ar), 7.22–7.12 (m, 5 H, Ar), 5.81 (d, 1 H, *J*_N*H*,2_ 9.5 Hz, N*H*), 5.55 (dd, 1 H, *J*_3,2_ 10.8, *J*_3,4_ 9.0 Hz, H‐3), 5.37–5.33 (m, 2 H, H‐4′, H‐5′), 5.21 (ddd, 1 H, *J*_7′,6’_ 9.6, *J*_7′,8′b_ 4.6, *J*_7′,8′a_ 2.5 Hz, H‐7′), 4.72 (d, 1 H, *J*_1,2_ 3.6 Hz, H‐1), 4.65 (d, 1 H, *J* 11.0 Hz, C*H*HPh), 4.59 (dd, 1 H, *J*_8′a,8′b_ 12.3 Hz, H‐8′a), 4.49 (d, 1 H, *J* 11.0 Hz, CH*H*Ph), 4.37 (ddd, 1 H, H‐2), 4.18 (dd, 1 H, *J*_6′,5’_ 1.1 Hz, H‐6′), 4.10 (dd, 1 H, H‐8′b), 3.92–3.88 (m, 1 H, H‐5), 3.76 (dd, 1 H, *J*_6a,6b_ 10.6, *J*_6a,5_ 1.7 Hz, H‐6a), 3.72 (s, 3 H, CO_2_C*H_3_*), 3.61 (app t, 1 H, *J*_4,5_ ∼9.7 Hz, H‐4), 3.57 (dd, 1 H, *J*_6b,5_ 7.2 Hz, H‐6b), 3.43 (s, 3 H, OC*H_3_*), 2.24–2.20 (m, 1 H, H‐3′*eq*), 2.10 (app t, 1 H, *J*_3′ax,3′eq_ ∼*J*_3′ax,4’_ 12.3 Hz, H‐3′*ax*), 2.08, 2.023, 2.020, 1.98, and 1.83 ppm (5 s, each 3 H, COC*H_3_*); ^13^C NMR: *δ*=170.5, 170.4, 170.0, 169.9, 169.7, 167.2 and 166.9 (7 s, 7C, 5×*C*OCH_3_, *C*OPh, C‐1′), 137.2 (s, 1C, Ar), 133.4 (d, 1C, Ar), 129.8 (d, 2C, Ar), 129.4 (s, 1C, Ar), 128.5 (d, 2C, Ar), 128.4 (d, 2C, Ar), 127.91 (d, 1C, Ar), 127.87 (d, 2C, Ar), 98.5 (s, C‐2′), 98.1 (d, C‐1), 76.6 (d, C‐4), 74.9 (t, *C*H_2_Ph), 74.3 (d, C‐3), 69.9 (d, C‐5), 68.6 (d, C‐6′), 67.7 (d, C‐7′), 66.3 (d, C‐4′), 64.5 (d, C‐5′), 63.2 (t, C‐6), 62.2 (t, C‐8′), 55.2 (q, O*C*H_3_), 52.6 (q, CO_2_ *C*H_3_), 52.4 (d, C‐2), 31.8 (t, C‐3′), 23.1, 20.8, 20.73, 20.69, and 20.6 ppm (5 q, 5C, CO*C*H_3_); HRMS (ESI‐TOF) *m/z* [*M*+Na]^+^ calcd for C_40_H_49_NO_18_Na^+^: 854.2842, found: 854.2847.

**Methyl (4,5,7,8‐tetra‐*O*‐acetyl‐3‐deoxy‐α‐d‐*manno*‐oct‐2‐ulopyranosyl)onate‐(2→6)‐methyl 2‐acetamido‐3‐*O*‐benzoyl‐2‐deoxy‐α‐d‐glucopyranoside (17)**: Compound **16** (70.0 mg, 0.084 mmol) was dissolved in dry MeOH (3.0 mL). The atmosphere was exchanged to argon by alternating evacuation and flushing with argon. Pd/C (10 %, 3 mg) was added followed by successive exchange of atmosphere to argon and hydrogen. After hydrogenation for 3 h, the mixture was filtered via a syringe filter, rinsed with MeOH (3×2 mL), and the filtrate was concentrated providing compound **17** as a colorless oil (60.0 mg, 96 %), which was used without purification: *R*_f_=0.43 (EtOAc); [α]_D_^20^=+99.3 (*c*=0.78 in CHCl_3_); ^1^H NMR (CDCl_3_): *δ*=8.05–8.03 (m, 2 H, Ar), 7.60–7.56 (m, 1 H, Ar), 7.47–7.43 (m, 2 H, Ar), 5.78 (d, 1 H, *J*_N*H*,2_ 9.8 Hz, N*H*), 5.37–5.32 (m, 2 H, H‐4′, H‐5′), 5.24 (dd, 1 H, *J*_3,2_ 10.7, *J*_3,4_ 9.2 Hz, H‐3), 5.23 (ddd, 1 H, *J*_7′,6’_ 9.3, *J*_7′,8′b_ 4.8, *J*_7′,8′a_ 2.6 Hz, H‐7′), 4.75 (d, 1 H, *J*_1,2_ 3.5 Hz, H‐1), 4.59 (dd, 1 H, *J*_8′a,8′b_ 12.1 Hz, H‐8′a), 4.45 (ddd, 1 H, H‐2), 4.28 (dd, 1 H, *J*_6′,5’_ 1.3 Hz, H‐6′), 4.15 (dd, 1 H, H‐8′b), 3.89–3.84 (m, 1 H, H‐5), 3.82 (s, 3 H, CO_2_C*H_3_*), 3.79–3.73 (m, 3 H, H‐4, H‐6a, H‐6b), 3.45 (s, 3 H, OC*H_3_*), 3.10 (d, 1 H, *J* 3.8 Hz, O*H*), 2.21–2.17 (m, 1 H, H‐3′*eq*), 2.12 (app t, 1 H, *J*_3′ax,3′eq_ ∼*J*_3′ax,4’_ 12.5 Hz, H‐3′*ax*), 2.085, 2.077, 2.00, 1.97, and 1.86 ppm (5 s, each 3 H, COC*H_3_*); ^13^C NMR (CDCl_3_): *δ*=170.6, 170.4, 169.91, 169.89, 169.8, 168.2, and 167.6 (7 s, 7C, 5×*C*OCH_3_, *C*OPh, C‐1′), 133.6 (d, 1C, Ar), 130.1 (d, 2C, Ar), 129.1 (s, 1C, Ar), 128.5 (d, 2C, Ar), 98.7 (s, C‐2′), 98.3 (d, C‐1), 75.5 (d, C‐3), 70.4 (d, C‐5), 70.0 (d, C‐4), 68.7 (d, C‐6′), 67.8 (d, C‐7′), 66.3 (d, C‐4′), 64.5 (d, C‐5′), 63.5 (t, C‐6), 62.3 (t, C‐8′), 55.3 (q, O*C*H_3_), 52.9 (q, CO_2_ *C*H_3_), 51.4 (d, C‐2), 31.9 (t, C‐3′), 23.2, 20.8, 20.73, 20.68, and 20.6 ppm (5 q, 5C, CO*C*H_3_); HRMS (ESI‐TOF) *m/z* [M+Na]^+^ calcd for C_33_H_43_NO_18_Na^+^: 764.2372, found: 764.2372.

**Methyl (4,5,7,8‐tetra‐*O*‐acetyl‐3‐deoxy‐α‐d‐*manno*‐oct‐2‐ulopyranosyl)onate‐(2→6)‐methyl 2‐acetamido‐3‐*O*‐benzoyl‐2‐deoxy‐4‐*O*‐(di‐benzylphosphoryl)‐α‐d‐glucopyranoside (18)**: A solution of compound **17** (19.5 mg, 0.026 mmol) in dry CH_2_Cl_2_ (1.0 mL) was degassed with argon. Under argon atmosphere 1 *H*‐tetrazole (5.5 mg, 0.079 mmol) was added followed by ground 4 Å molecular sieves (50 mg). After 30 min the suspension was cooled to 0 °C and treated dropwise with dibenzyl *N,N*‐diisopropylphosphoramidite (17.3 μL, 0.053 mmol, in 3 portions within 70 min). *m*CPBA (70 w %, 9.7 mg, 0.039 mmol) was added at 0 °C, and the mixture was stirred for 1 h. The mixture was diluted with CH_2_Cl_2_ and satd. NaHCO_3_, the aqueous phase was extracted with CH_2_Cl_2_ (3×5mL), and the combined organic layers were dried over MgSO_4_. Filtration, concentration of the organic phase, and chromatography of the residue (toluene/EtOAc 1:4) provided phosphorylated compound **18** with a minor impurity which was separated by HP‐column chromatography (*n*‐hexane/EtOAc 1:2→1:4) affording **18** as a colorless oil (18.3 mg, 70 %): *R*_f_=0.41 (*n*‐hexane/EtOAc 1:4); [α]_D_^20^=+64.8 (*c*=0.78 in CHCl_3_); ^1^H NMR (CDCl_3_, ref. to 0.00, TMS): *δ*=8.03–8.01 (m, 2 H, Ar), 7.54 −7.51 (m, 1 H, Ar), 7.39–7.35 (m, 2 H, Ar), 7.29–7.13 (m, 8 H, Ar), 6.91–6.89 (m, 2 H, Ar), 5.79 (d, 1 H, *J*_N*H*,2_ 9.4 Hz, N*H*), 5.56 (dd, 1 H, *J*_3,2_ 10.9, *J*_3,4_ 9.1 Hz, H‐3), 5.41 (ddd, 1 H, *J*_4′,3′ax_ 12.2, *J*_4′,3′eq_ 5.1, *J*_4′,5’_ 3.1 Hz, H‐4′), 5.37–5.35 (m, 1 H, H‐5′), 5.21 (ddd, 1 H, *J*_7′,6’_ 9.2, *J*_7′,8′b_ 5.0, *J*_7′,8′a_ 2.4 Hz, H‐7′), 4.89 (dd, 1 H, ^2^ *J* 11.8, *J*_H,P_ 7.2 Hz, C*H*HPh), 4.78 (dd, 1 H, ^2^ *J* 12.2, *J*_H,P_ 7.9 Hz, C*H*HPh), 4.77 (d, 1 H, *J*_1,2_ 3.8 Hz, H‐1), 4.74 (dd, 1 H, ^2^ *J* 11.7, *J*_H,P_ 7.5 Hz, CH*H*Ph), 4.67 (dd, 1 H, *J*_8′a,8′b_ 12.2 Hz, H‐8′a), 4.56 (dd, 1 H, ^2^ *J* 11.8, *J*_H,P_ 10.0 Hz, CH*H*Ph), 4.51 (app q, 1 H, *J*_4,5_ ∼*J*_4,P_ 9.3 Hz, H‐4), 4.41 (ddd, 1 H, H‐2), 4.34 (dd, 1 H, *J*_6′,5’_ 1.4 Hz, H‐6′), 4.09 (dd, 1 H, H‐8′b), 4.05–4.00 (m, 2 H, H‐5, H‐6a), 3.74 (dd, 1 H, *J*_6b,6a_ 11.1, *J*_6b,5_ 7.9 Hz, H‐6b), 3.68 (s, 3 H, CO_2_C*H_3_*), 3.48 (s, 3 H, OC*H_3_*), 2.22–2.18 (m, 1 H, H‐3′*eq*), 2.12 (app t, 1 H, *J*_3′ax,3′eq_ ∼12.5 Hz, H‐3′*ax*), 2.091, 2.086, 2.01, 1.96, and 1.84 ppm (5 s, each 3 H, COC*H_3_*); ^13^C NMR (CDCl_3_): *δ*=170.5, 170.4, 169.9, 169.83, 169.80, 167.2, and 166.9 (7 s, 7C, 5×*C*OCH_3_, *C*OPh, C‐1′), 135.5 (ds, 1C, *J* 7.2 Hz, Ar), 135.2 (ds, 1C, *J* 6.5 Hz, Ar), 133.4 and 130.0 (2 d, 3C, Ar), 129.1 (s, 1C, Ar), 128.44, 128.39, 128.32, 128.30, 127.69, and 127.66 (6 d, 12C, Ar), 98.5 (s, C‐2′), 97.8 (d, C‐1), 73.8 (dd, *J* 5.9 Hz, C‐4), 72.1 (dd, *J* 2.0 Hz, C‐3), 69.53 (d, C‐5), 69.47 (dt, *J* 5.8 Hz, *C*H_2_Ph), 69.4 (dt, *J* 5.9 Hz, *C*H_2_Ph), 68.8 (d, C‐6′), 68.1 (d, C‐7′), 66.3 (d, C‐4′), 64.7 (d, C‐5′), 62.7 (t, C‐6), 62.1 (t, C‐8′), 55.4 (q, O*C*H_3_), 52.6 (q, CO_2_ *C*H_3_), 52.2 (d, C‐2), 31.9 (t, C‐3′), 23.1, 20.8, 20.73, and 20.66 ppm (4 s, 5C, CO*C*H_3_); ^31^P NMR (CDCl_3_): *δ*=−2.01 ppm; HRMS (ESI‐TOF) *m/z* [M+Na]^+^ calcd for C_47_H_56_NO_21_PNa^+^: 1024.2975, found: 1024.2976.

**Sodium (3‐deoxy‐α‐d‐*manno*‐oct‐2‐ulopyranosyl)onate‐(2→6)‐methyl 2‐acetamido‐2‐deoxy‐α‐d‐glucopyranoside (19)**: A solution of **17** (8.2 mg, 0.011 mmol) in dry MeOH (1 mL) was treated with sodium methoxide (0.1 m in MeOH, 22 μL, 2.2 μmol) at ambient temperature for 3 h. Ion‐exchange resin DOWEX 50 (H^+^‐form) was added until the mixture reacted neutral. The resin was filtered off and the filtrate was concentrated in vacuo. To remove methyl benzoate, water (3 mL) and *n*‐hexane (3 mL) were added to the residual solid, the mixture was ultrasonicated for 10 s, and the organic layer was removed. Fresh *n*‐hexane (3 mL) was added and the procedure was repeated. After two more extractions, the aqueous phase was concentrated in vacuo, and the residue was stirred in aq NaOH (0.01 m, 3.0 mL) at 0 °C for 3 h. Additional aq NaOH (0.1 m, 0.5 mL) was added, and after 2 h at 0 °C the mixture was neutralized with DOWEX 50 (H^+^‐form). The resin was filtered off, washed with water (3×1 mL), and the filtrate was freeze‐dried. The crude product was desalted on a PD10 SEC column (H_2_O). Freeze‐drying of pooled fractions yielded sodium salt **19** as a colorless amorphous solid (5.1 mg, 97 %): [α]_D_^20^=+102.8 (*c*=0.26 in H_2_O); ^1^H NMR (D_2_O, pD 7.4): *δ*=4.71 (d, 1 H, *J*_1,2_ 3.7 Hz, H‐1, overlapped by residual solvent peak), 4.04 (ddd, 1 H, *J*_4′,3′ax_ 12.1, *J*_4′,3′eq_ 5.0, *J*_4′,5’_ 2.9 Hz, H‐4′), 3.99–3.97 (m, 1 H, H‐5′), 3.92–3.87 (m, 3 H, H‐2, H‐7′, H‐8′a), 3.74 (ddd, 1 H, *J*_5,4_ 10.1, *J*_5,6a_ 4.5, *J*_5,6b_ 3.4 Hz, H‐5), 3.68–3.54 (m, 5 H, H‐3, H‐6a, H‐6b, H‐6′, H‐8′b), 3.44 (dd, 1 H, *J*_4,3_ 9.4 Hz, H‐4), 3.34 (s, 3 H, OC*H_3_*), 2.05 (dd, 1 H, *J*_3′eq,3′ax_ 13.1 Hz, H‐3′*eq*), 1.99 (s, 3 H, COC*H_3_*), and 1.76 ppm (app t, 1 H, H‐3′*ax*); ^13^C NMR (D_2_O, pD 7.4): *δ*=175.8 and 175.3 (2 s, 2C, C‐1′, *C*OCH_3_), 100.7 (s, C‐2′), 98.8 (d, C‐1), 72.3 and 72.2 (2 d, 2C, C‐3, C‐6′), 71.2 (d, C‐4), 71.0 (d, C‐5), 70.3 (d, C‐7′), 67.1 and 66.9 (2 d, 2C, C‐4′, C‐5′), 64.2 (t, C‐8′), 62.7 (t, C‐6), 56.0 (q, O*C*H_3_), 54.4 (d, C‐2), 34.9 (t, C‐3′), and 22.7 ppm (q, CO*C*H_3_); HRMS (ESI‐TOF) *m/z* [M−H]^−^ calcd for C_17_H_28_NO_13_^−^: 454.1555, found: 454.1564.

**3‐Deoxy‐α‐d‐*manno*‐oct‐2‐ulopyranosylonic acid‐(2→6)‐methyl 2‐acetamido‐2‐deoxy‐4‐phosphono‐α‐d‐glucopyranoside sodium salt (20)**: Phosphotriester **18** (11.0 mg, 0.011 mmol) was dissolved in dry MeOH (2.0 mL). The atmosphere was exchanged to argon by alternating evacuation and flushing with argon. Pd/C (10 %, 1 mg) was added followed by successive exchange of atmosphere to argon and hydrogen. After hydrogenation for 30 min the mixture was filtered via a syringe filter into a flask containing sodium methoxide (0.1 m in MeOH, 110 μL, 0.011 mmol) in dry MeOH (2.0 mL). After rinsing the filter with MeOH (3×1 mL), the filtrate was neutralized by further addition of sodium methoxide (0.1 m in MeOH). The solvent was removed until a volume of 3 mL of MeOH remained: The pH was adjusted to 8 with sodium methoxide, and the mixture was stirred for 3 h at rt. After increasing the pH to 9, stirring was continued for 14 h. Excessive base was neutralized by adding DOWEX 50 (H^+^‐form) resin. The resin was filtered off, rinsed with MeOH, and the filtrate was concentrated in vacuo. Removal of methyl benzoate, ester saponification, and desalting were performed as described for compound **19**, providing phosphate **20** as a colorless amorphous solid (6.3 mg, 99 %): [α]_D_^20^=+91.6 (*c*=0.63 in H_2_O); ^1^H NMR (D_2_O, pD 7.4): *δ*=4.71 (d, 1 H, *J*_1,2_ 3.5 Hz, H‐1, overlapped by residual solvent peak), 4.06 (ddd, 1 H, *J*_4′,3′ax_ 12.0, *J*_4′,3′eq_ 5.2, *J*_4′,5’_ 3.0 Hz, H‐4′), 3.99–3.98 (m, 1 H, H‐5′), 3.92–3.76 (m, 7 H, H‐2, H‐3, H‐4, H‐5, H‐6′, H‐7′, H‐8′a), 3.70–3.63 (m, 2 H, H‐6a, H‐8′b), 3.57–3.53 (m, 1 H, H‐6b), 3.34 (s, 3 H, OC*H_3_*), 2.03 (dd, 1 H, *J*_3′eq,3′ax_ 13.1 Hz, H‐3′*eq*), 1.98 (s, 3 H, COC*H_3_*), and 1.74 ppm (app t, 1 H, H‐3′*ax*); ^13^C NMR (D_2_O, pD 7.4): *δ*=176.0 and 175.1 (2 s, 2C, C‐1′, *C*OCH_3_), 100.6 (s, C‐2′), 98.1 (d, C‐1), 74.3 (dd, *J* 5.2 Hz, C‐4), 72.2 (d, 2C, C‐3, C‐6′), 70.5 (d, C‐7′), 70.3 (dd, *J* 8.7 Hz, C‐5), 67.2 (d, C‐5′), 66.9 (d, C‐4′), 64.2 (t, C‐8′), 63.2 (t, C‐6), 55.9 (q, O*C*H_3_), 54.1 (d, C‐2), 35.0 (t, C‐3′), and 22.7 ppm (q, CO*C*H_3_); ^31^P NMR (D_2_O, pD 7.4): *δ*=−3.30 ppm; HRMS (ESI‐TOF) *m/z* [M−H]^−^ calcd for C_17_H_29_NO_16_P^−^: 534.1219, found: 534.1229.

## Supporting information

As a service to our authors and readers, this journal provides supporting information supplied by the authors. Such materials are peer reviewed and may be re‐organized for online delivery, but are not copy‐edited or typeset. Technical support issues arising from supporting information (other than missing files) should be addressed to the authors.

Supplementary
