# Supplementary material for: Scope and Limitations of 3‐Iodo‐Kdo Fluoride‐Based Glycosylation Chemistry using N‐Acetyl Glucosamine Acceptors
Source: ChemistryOpen. 2015 Jul 29;4(6):722–8. doi: 10.1002/open.201500126 (PMC4906502; doi:10.1002/open.201500126)
Supplement: Supplementary file 1 — Supplementary [file OPEN-4-722-s001.pdf]

ChemistryOPEN

Supporting Information

© 2015 The Authors. Published by Wiley-VCH Verlag GmbH & Co. KGaA, Weinheim

Scope and Limitations of 3-Iodo-Kdo Fluoride-Based Glycosylation Chemistry using *N*-Acetyl Glucosamine Acceptors**

Barbara Pokorny and Paul Kosma*[a]

open_201500126_sm_miscellaneous_information.pdf

Supporting information

Table of contents

1. Table of contents S-1

2. Synthesis of glucosamine acceptors S-2

3. Synthetic details for compounds **3**, **4**, **5**, **11**, **12** and **13** S-4

4. NMR studies on competitive iodonium ion migration S-11

5. NMR spectra of compounds **3** – **5** and **15** – **20** S-17

6. References S-29

2. Synthesis of glucosamine acceptors

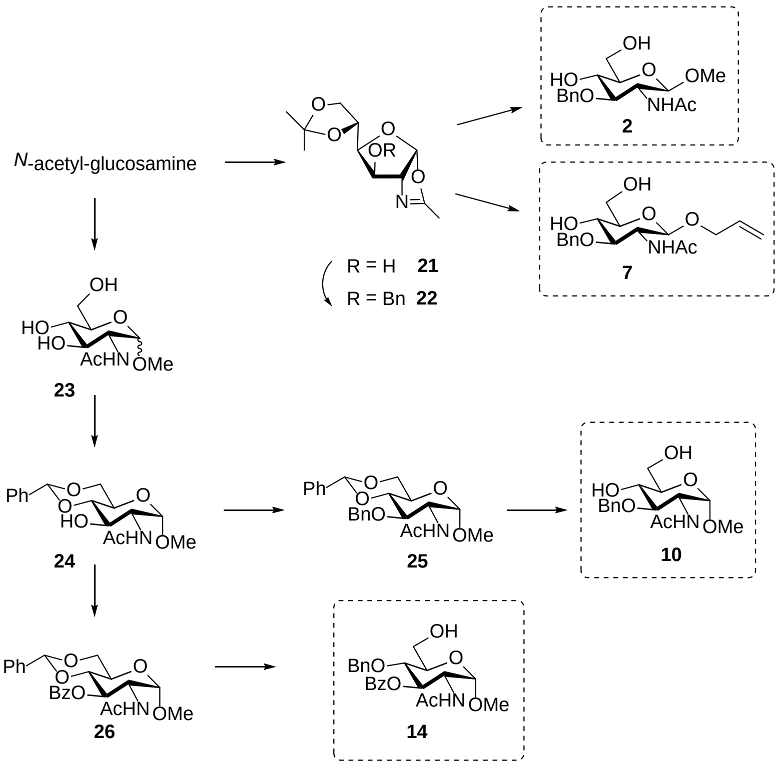

2.1. Methyl 2-acetamido-3-*O*-benzyl-2-deoxy-β-D-glucopyranoside (2)

Starting from *N*-acetyl glucosamine, acceptor **2** was prepared in three steps according to literature.[S1]

2.2. Allyl 2-acetamido-3-*O*-benzyl-2-deoxy-β-D-glucopyranoside (7)

The 3-*O*-benzylated oxazoline **22**[S1b] (0.51 g, 1.52 mmol) was reacted with allyl alcohol (17 mL) in the presence of camphorsulfonic acid (0.10 g, 0.42 mmol) similar to the literature procedure for compound **2**. The crude product was purified by two consecutive chromatography steps ($CHCl_3$:MeOH 95:5; EtOAc:EtOH 95:5) affording pure β-allyl glycoside **7**[S2] (0.21 g, 37%): ^{1}H NMR (CD_3OD) = δ 7.34 - 7.23 (m, 5H, Ar), 5.88 (dddd, 1H, *J* 17.3, *J* 10.6, *J* 5.7, *J* 4.9 Hz, CH_2-C*H*=CH_2), 5.29 - 5.25 (m, 1H, CH_2-CH=C*H*H), 5.15 - 5.12 (m, 1H, CH_2-CH=CH*H*), 4.86 (d, 1H, *J* 11.3 Hz, C*H*HPh, overlapped by water peak), 4.65 (d, 1H, *J* 11.6 Hz, CH*H*Ph), 4.48 (d, 1H, $J_{1,2}$ 8.4 Hz, H-1), 4.33 (tdd, 1H, *J* 13.3, *J* 4.9, *J* 1.6 Hz, C*H*H-CH=CH_2), 4.07 (tdd, 1H, *J* 13.3, *J* 5.8, *J* 1.6 Hz, CH*H*-CH=CH_2), 3.89 (dd, 1H, $J_{6a,6b}$ 11.9, $J_{6a,5}$ 2.3 Hz, H-6a), 3.81 - 3.75 (m, 1H, H-2), 3.69 (dd, 1H, $J_{6b,5}$ 6.1 Hz, H-6b), 3.53 - 3.48 (m, 2H, H-3, H-4), 3.32 - 3.27 (m, 1H, H-5), 1.88 ppm (s, 3H, COCH_3).

2.3. Methyl 2-acetamido-3-*O*-benzyl-2-deoxy-α-D-glucopyranoside (10)

A mixture of *N*-acetyl-D-glucosamine (5.00 g, 22.6 mmol) in dry MeOH (50 mL) containing ion exchange resin DOWEX 50 (H^+ form, 5 g) was heated to reflux for 18 h. The cooled mixture was filtered and rinsed with MeOH. The filtrate was concentrated providing an α/β-mixture of **23** (5.25 g, 97%, α:β = 10:1). According to literature[S3] this mixture was used to prepare **25**, and the β-anomer was removed by crystallization of the intermediate **24**. The benzylidene group of compound **25** was cleaved according to literature[S4]. The ^{1}H NMR spectrum of **10** was in agreement with data in the literature[S3].

2.4. Methyl 2-acetamido-3-*O*-benzoyl-4-*O*-benzyl-2-deoxy-α-D-glucopyranoside (14)

Compound **24**[S3] (175 mg, 0.54 mmol) was dissolved in dry pyridine (3.5 mL) and treated with benzoyl chloride (187 µL, 1.62 mmol) at 0 °C. After stirring for 16 h at ambient temperature excessive reagent was destroyed by slow addition of dry MeOH (2 mL, at 0 °C). The mixture was coevaporated with toluene (2x) and the residue was purified by chromatography (toluene/EtOAc 1:1) yielding **26**[S5] (165 mg, 71%). A solution of compound **26** (61 mg, 0.143 mmol) in dry CH_2Cl_2 (3.0 mL) containing 4 Å ground molecular sieves (150 mg) was stirred at ambient temperature for 1 h. To the cooled (-78 °C) mixture triethylsilane (114 µL, 0.714 mmol) and dichlorophenylborane (93 µL, 0.714 mmol) were added dropwise.[S6] After 30 min triethylamine (0.35 mL) and dry MeOH (0.35 mL) were added consecutively. The mixture was partitioned between chloroform and satd. $NaHCO_3$, the aqueous phase was further extracted (2x) with chloroform. The combined organic phases were dried ($MgSO_4$), filtered and the filtrate was concentrated. The residue was purified by two consecutive chromatographic separations (EtOAc:EtOH 9:1, then $CHCl_3$:MeOH 100:2) which gave acceptor **14** (43 mg, 70%); the analytical data was in agreement with published data.[S7]

2.5. Allyl 3-*O*-benzyl-2-deoxy-2-phthalimido-β-D-glucopyranoside (8)

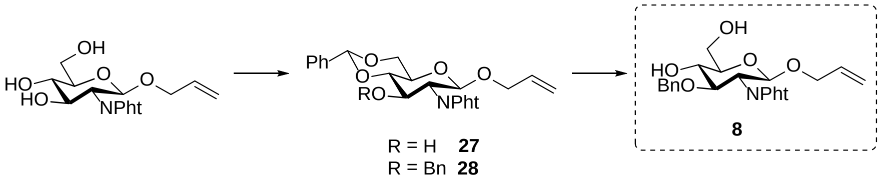

A solution of allyl 2-deoxy-2-phthalimido-β-glucopyranoside (140 mg, 0.40 mmol), benzaldehyde dimethyl acetal (72 µL 0.48 mmol) and *p*-toluenesulfonic acid monohydrate (4 mg, 0.024 mmol) in dry acetonitrile (6.0 mL) was stirred at ambient temperature for 1 h. After addition of triethylamine (12 µL) the volatile components were removed in *vacuo* and the residue was purified by chromatography (toluene/EtOAc 40:1 →7:1) affording **27**[S8] (162 mg, 92%): ^{1}H NMR ($CDCl_3$) = δ 7.88 - 7.34 (m, 9H, Ar), 5.72 - 5.65 (m, 1H, CH_2-C*H*=CH_2), 5.56 (s, 1H, C*H*Ph), 5.29 (d, 1H, $J_{1,2}$ 8.5 Hz, H-1), 5.15 - 5.11 (m, 1H, CH_2-CH=C*H*H), 5.06 - 5.03 (m, 1H, CH_2-CH=CH*H*), 4.65 - 4.60 (m, 1H, H-3), 4.40 - 4.36 (m, 1H, H-6a), 4.30 - 4.24 (m, 2H, H-2, C*H*H-CH=CH_2), 4.05 - 4.00 (m, 1H, CH*H*-CH=CH_2), 3.83 (app t, 1H, $J_{6b,6a}$ ~ $J_{6b,5}$ 10.0 Hz, H-6b), 3.65 - 3.58 (m, 2H, H-4, H-5) and 2.68 ppm (d, 1H, *J* 4.0 Hz, O*H*).

A solution of compound **27** (346 mg, 0.79 mmol) in dry DMF (10.0 mL) was treated with sodium hydride (60% in mineral oil, 63 mg, 1.58 mmol) portionwise and was stirred for 30 min after complete addition. Next, benzyl bromide (376 µL, 3.16 mmol) was added and stirring was continued for and 1 h at room temperature. Excessive reagent was destroyed by addition of dry MeOH (2 mL). The mixture was partitioned between satd. ammonium chloride and diethyl ether and the aqueous phase was extracted with diethyl ether twice. The combined organic layers were dried ($MgSO_4$), filtered and concentrated. The residue was purified by chromatography (toluene/EtOAc 20:1) providing **28**[S9] (294 mg, 71%): ^{1}H NMR ($CDCl_3$) = δ 7.90 - 7.36 (m, 9H, Ar), 7.02 - 6.86 (m, 5H, Ar), 5.69 - 5.63 (m, 1H, CH_2-C*H*=CH_2), 5.62 (s, 1H, C*H*Ph), 5.25 (d, 1H, $J_{1,2}$ 8.4 Hz, H-1), 5.13 - 5.09 (m, 1H, CH_2-CH=C*H*H), 5.03 - 5.00 (m, 1H, CH_2-CH=CH*H*), 4.80 (d, 1H, *J* 12.4 Hz, C*H*HPh), 4.51 (d, 1H, *J* 12.3 Hz, CH*H*Ph), 4.44 (dd, 1H, $J_{3,2}$ 10.2, $J_{3,4}$ 8.8 Hz, H-3), 4.41 (dd, 1H, $J_{6a,6b}$ 10.1, $J_{6a,5}$ 4.6 Hz, H-6a), 4.28 - 4.23 (m, 2H, H-2, C*H*H-CH=CH_2), 4.02 - 3.98 (m, 1H, CH*H*-CH=CH_2), 3.87 (app t, 1H, $J_{6b,5}$ ~ 9.8 Hz, H-6b), 3.83 (app t, 1H, $J_{4,5}$ ~ 9.3 Hz, H-4) and 3.65 ppm (app td, 1H, H-5).

Compound **28** (290 mg, 0.55 mmol) was dissolved in 80% AcOH (10 mL) and heated to 60 °C for 2.5 h. The mixture was concentrated and purified by chromatography (toluene/EtOAc 1:1) yielding **8**[S10] (230 mg, 95%): ^{1}H NMR ($CDCl_3$) = δ 7.86 - 7.64 (m, 4H, Ar), 7.09 - 6.93 (m, 5H, Ar), 5.67 (dddd, 1H, *J* 17.2, *J* 10.4, *J* 6.2, *J* 5.2 Hz, CH_2-C*H*=CH_2), 5.21 (d, 1H, $J_{1,2}$ 8.7 Hz, H-1), 5.12 - 5.07 (m, 1H, CH_2-CH=C*H*H), 5.03 - 5.00 (m, 1H, CH_2-CH=CH*H*), 4.70 (d, 1H, *J* 12.4 Hz, C*H*HPh), 4.54 (d, 1H, *J* 12.2 Hz, CH*H*Ph), 4.28 (dd, 1H, $J_{3,2}$ 10.7, $J_{3,4}$ 8.6 Hz, H-3), 4.24 - 4.21 (m, 1H, C*H*H-CH=CH_2), 4.18 (dd, 1H, H-2), 4.02 - 3.98 (m, 1H, CH*H*-CH=CH_2), 3.95 (dd, 1H, $J_{6a,6b}$ 11.8, $J_{6a,5}$ 3.6 Hz, H-6a), 3.88 (dd, 1H, $J_{6b,5}$ 4.1 Hz, H-6b), 3.82 (app td, 1H, $J_{4,5}$ 9.7, $J_{4,OH}$ 3.1 Hz, H-4), 3.53 (ddd, 1H, H-5) and 2.85 ppm (d, 1H, *J* 3.7 Hz, O*H*).

3. Synthetic details for compounds (3), (4), (5), (11), (12) and (13)

3.1. Methyl (4,5,7,8-tetra-*O*-acetyl-3-deoxy-3-iodo-D-*glycero*-α-D-*talo*-oct-2-ulopyranosyl)onate-(2→6)-methyl 2-acetamido-3-*O*-benzyl-2-deoxy-β-D-glucopyranoside (3) and 2-methyl [methyl (4,5,7,8-tetra-*O*-acetyl-3-deoxy-3-iodo-D-*glycero*-α-D-*talo*-oct-2-ulopyranosyl)onate-(2→6)-3-*O*-benzyl-1,2-dideoxy-α-D-glucofurano]-[2,1-d]-2-oxazoline (4) and [methyl (4,5,7,8-tetra-*O*-acetyl-3-deoxy-3-iodo-D-*glycero*-α-D-*talo*-oct-2-ulopyranosyl)onate-(2→6)-3-*O*-benzyl-1,2-dideoxy-α-D-glucopyrano]-[2,1-d]-2-oxazoline (5).

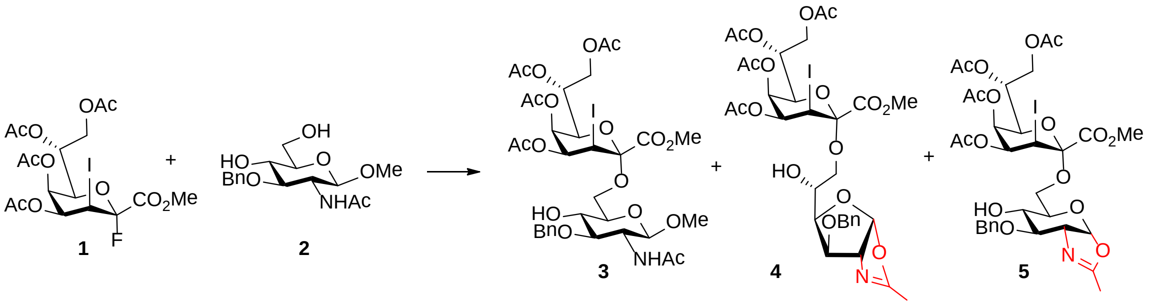

A suspension of glycosyl acceptor **2** (28.3 mg, 0.087 mmol) and 3-iodo donor **1** (52.5 mg, 0.096 mmol) in dry CH_2Cl_2 (4.0 mL) containing ground 3 Å molecular sieves (200 mg) was stirred at ambient temperature for 2h. $BF_3·Et_2O$ (35.7 µL, 0.278 mmol) was added at 0 °C and the mixture kept at ambient temperature for 90 min. After addition of satd. aq. $NaHCO_3$ and CH_2Cl_2, the mixture was extracted, the aqueous phase once again treated with CH_2Cl_2 and the combined organic layers were washed successively with sodium thiosulfate (5 w%) and brine. The organic layer was dried ($MgSO_4$), filtered and concentrated. The crude product was purified by chromatography (toluene/EtOAc 2:1→0:1) affording a mixture of several disaccharide compounds, which were further separated by HP-chromatography (EtOAc) which afforded disaccharide **3** (23.5 mg, 32%), furano-oxazoline **4** (7.6 mg, 11%) and pyrano-oxazoline **5** (2.1 mg, 3 %) as colourless oils.

3: $[\alpha]_D^{20}$ +37.2 (*c* = 0.72, $CHCl_3$); R_f 0.44 (EtOAc, HP-TLC); ^{1}H NMR ($CDCl_3$) = δ 7.38 - 7.29 (m, 5H, Ar), 5.66 (d, 1H, $J_{NH,2}$ 7.9 Hz, N*H*), 5.38 - 5.35 (m, 2H, H-5', H-7'), 5.02 (dd, 1H, $J_{4',3'}$ 4.7, $J_{4',5'}$ 3.7 Hz, H-4'), 4.73 (d, 1H, *J* 11.9 Hz, C*H*HPh), 4.71 (d, 1H, $J_{1,2}$ 8.4 Hz, H-1), 4.68 (d, 1H, *J* 11.7 Hz, CH*H*Ph), 4.64 (dd, 1H, $J_{8'a,8'b}$ 12.2, $J_{8'a,7'}$ 2.3 Hz, H-8'a), 4.50 (dd, 1H, $J_{3',5'}$ 0.7 Hz, H-3'), 4.44 (dd, 1H, $J_{6',7'}$ 9.7, $J_{6',5'}$ 1.8 Hz, H-6'), 4.19 (dd, 1H, $J_{8'b,7'}$ 4.5 Hz, H-8'b), 3.95 (dd, 1H, $J_{3,2}$ 10.2, $J_{3,4}$ 8.7 Hz, H-3), 3.84 (s, 3H, CO_2CH_3), 3.68 (dd, 1H, $J_{6a,6b}$ 10.1, $J_{6a,5}$ 6.9 Hz, H-6a), 3.58 (dd, 1H, $J_{6b,5}$ 2.5 Hz, H-6b), 3.51 (ddd, 1H, $J_{5,4}$ 9.6 Hz, H-5), 3.47 (s, 3H, OC*H*$_3$), 3.41 (app dt, 1H, $J_{4,OH}$ 3.1 Hz, H-4), 3.30 (app td, 1H, H-2), 2.59 (d, 1H, O*H*), 2.11, 2.05, 2.04, 1.96 and 1.94 ppm (5 s, each 3H, COC*H*$_3$); ^{13}C NMR ($CDCl_3$) = δ 170.69, 170.65, 170.2, 169.5 and 169.4 (5 s, 5C, COC*H*$_3$), 166.1 (s, C-1'), 138.1 (s, 1C, Ar), 128.7 (d, 2C, Ar), 128.2 (d, 1C, Ar), 128.1 (d, 2C, Ar), 101.3 (s, C-2'), 100.9 (d, C-1), 80.7 (d, C-3), 74.2 (t,

CH_2Ph), 73.9 (d, C-5), 71.4 (d, C-4), 68.1 (d, C-6'), 67.7 (d, C-7'), 65.5 (t, C-6), 65.4 (d, C-4'), 63.3 (d, C-5'), 62.0 (t, C-8'), 57.1 (d, C-2), 56.9 (q, OCH_3), 53.1 (q, CO_2CH_3), 23.6 (q, $COCH_3$), 21.9 (d, C-3'), 20.90, 20.86, 20.7 and 20.6 ppm (4 q, 4C, $COCH_3$); HRMS (ESI-TOF): *m/z* calcd for $C_{33}H_{44}INO_{17}Na^+$: 876.1546 [M+Na$^+$]; found: = 876.1532.

4: R_f 0.42 (EtOAc, HP-TLC); ^{1}H NMR ($CDCl_3$) = δ 7.40 - 7.30 (m, 5H, Ar), 6.13 (d, 1H, $J_{1,2}$ 5.2 Hz, H-1), 5.41 – 5.36 (m, 2H, H-5', H-7'), 4.98 (dd, 1H, $J_{4',3'}$ 4.8, $J_{4',5'}$ 3.6 Hz, H-4'), 4.76 (d, 1H, *J* 12.0 Hz, C*H*HPh), 4.63 - 4.56 (m, 3H, H-2, H-8'a, CH*H*Ph), 4.50 (dd, 1H, $J_{3',5'}$ 0.6 Hz, H-3'), 4.43 (dd, 1H, $J_{6',7'}$ 9.8, $J_{6',5'}$ 2.1 Hz, H-6'), 4.23 (dd, 1H, $J_{8'b,8'a}$ 12.5, $J_{8'b,7'}$ 4.4 Hz, H-8'b), 4.14 - 4.09 (m, 2H, H-3, H-5), 3.82 (s, 3H, CO_2CH_3), 3.77 (dd, 1H, $J_{4,5}$ 8.6, $J_{4,3}$ 3.2 Hz, H-4), 3.73 (dd, 1H, $J_{6a,6b}$ 10.0, $J_{6a,5}$ 5.5 Hz, H-6a), 3.42 (dd, 1H, $J_{6b,5}$ 2.6 Hz, H-6b), 2.33 (b d, 1H, *J* 6.8 Hz, O*H*), 2.12 (s, 3H, $COCH_3$), 2.06 (s, 3H, $COCH_3$), 2.04 (d, 3H, *J* 1.4 Hz, oxazoline-CH_3), 2.01 (s, 3H, $COCH_3$), 1.98 ppm (s, 3H, $COCH_3$); ^{13}C NMR ($CDCl_3$) = δ 170.6, 170.2, 169.6 and 169.2, 167.3 [s, N=*C*(CH_3)O], 166.2 (s, C-1'), 137.1 (s, 1C, Ar), 128.8 (d, 2C, Ar), 128.3 (d, 1C, Ar), 127.9 (d, 2C, Ar), 106.9 (d, C-1), 101.5 (s, C-2'), 81.2 (d, C-3), 79.6 (d, C-4), 74.9 (d, C-2), 71.9 (t, CH_2Ph), 68.2 (d, C-6'), 67.7 (d, C-7'), 67.6 (t, C-6), 67.1 (d, C-5), 65.3 (d, C-4'), 63.3 (d, C-5'), 62.0 (t, C-8'), 53.2 (q, CO_2CH_3), 21.8, 20.9, 20.8, 20.7 and 20.6 (4 q, 1d, 5C, C-3', 4 x $COCH_3$), 14.0 (q, oxazoline-CH_3) ppm; HRMS (ESI-TOF): *m/z* calcd for $C_{32}H_{40}INO_{16}H^+$: 822.1465 [M+H$^+$]; found: 822.1458.

5: R_f 0.38 (EtOAc, HP-TLC); ^{1}H NMR ($CDCl_3$) = δ 7.38 - 7.28 (m, 5H, Ar), 5.96 (d, 1H, $J_{1,2}$ 7.2 Hz, H-1), 5.45 - 5.43 (m, 1H, H-5'), 5.36 (ddd, 1H, $J_{7',6'}$ 9.5, $J_{7',8'b}$ 4.3, $J_{7',8'a}$ 2.4 Hz, H-7'), 5.03 (dd, 1H, $J_{4',3'}$ 4.7, $J_{4',5'}$ 3.9 Hz, H-4'), 4.78 (d, 1H, *J* 12.0 Hz, C*H*HPh), 4.65 (d, 1H, *J* 11.8 Hz, CH*H*Ph), 4.64 (dd, 1H, $J_{8'a,8'b}$ 12.3 Hz, H-8'a), 4.54 (b d, 1H, H-3'), 4.42 (dd, 1H, $J_{6',5'}$ 2.0 Hz, H-6'), 4.21 (dd, 1H, H-8'b), 4.12 - 4.08 (m, 1H, H-2), 3.85 (s, 3H, CO_2CH_3), 3.75 (dd, 1H, $J_{6a,6b}$ 10.0, $J_{6a,5}$ 5.9 Hz, H-6a), 3.71 (app t, 1H, $J_{3,2} = J_{3,4}$ 4.4 Hz, H-3), 3.66 - 3.62 (m, 1H, H-4), 3.49 (ddd, 1H, $J_{5,4}$ 8.8, $J_{5,6b}$ 2.7 Hz, H-5), 3.45 (dd, 1H, H-6b), 2.31 - 2.25 (m, 1H, O*H*), 2.12 (s, 3H, $COCH_3$), 2.08 (d, 3H, *J* 1.6 Hz, oxazoline-CH_3), 2.06 (s, 3H, $COCH_3$), 1.99 (s, 3H, $COCH_3$), 1.97 ppm (s, 3H, $COCH_3$); ^{13}C NMR ($CDCl_3$) = δ 170.7, 170.2, 169.6 and 169.3 (4 s, 4C, *C*OCH_3), 166.8 [s, N=*C*(CH_3)-O], 166.0 (s, C-1'), 137.5 (s, 1C, Ar), 128.6 (d, 2C, Ar), 128.04 (d, 1C, Ar), 127.96 (d, 2C, Ar), 101.5 (s, C-2'), 101.1 (d, C-1), 80.4 (d, C-3), 72.2 (d, C-5), 72.0 (t, CH_2Ph), 68.3 and 68.2 (2 d, 2C, C-4, C-6'), 67.8 (d, C-7'), 66.3 (d, C-2), 66.1 (t, C-6), 65.4 (d, C-4'), 63.4 (d, C-5'), 61.9 (t, C-8'), 53.1 (q, CO_2CH_3), 21.9, 20.9, 20.8, 20.7 and 20.6 (4 q, 1 d, 5C, C-3', 4 x $COCH_3$), 14.3 (q, oxazoline-CH_3) ppm; HRMS (ESI-TOF): *m/z* calcd for $C_{32}H_{40}INO_{16}H^+$: 822.1465 [M+H$^+$]; found: 822.1483.

3.2. Methyl (4,5,7,8-tetra-*O*-acetyl-3-deoxy-3-iodo-D-*glycero*-α-D-*talo*-oct-2-ulopyranosyl)onate-(2→6)-methyl 2-acetamido-3-*O*-benzyl-2-deoxy-α-D-glucopyranoside (11)

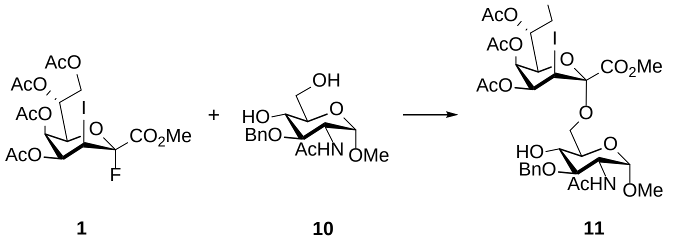

1 **10** **11**

A solution of donor **1** (40 mg, 0.074 mmol) in dry CH_2Cl_2 (3.0 mL) was added to acceptor **10** (20 mg, 0.061 mmol) and the suspension was stirred at ambient temperature for 1 h in the presence of 3 Å ground molecular sieves (150 mg). $BF_3.Et_2O$ (23 µL, 0.184 mmol) was added at 0 °C. After 1 h at room temperature satd. aq. $NaHCO_3$ solution was added and the mixture was repeatedly extracted with

CH_2Cl_2. The combined organic layers were washed successively with thiosulfate (5 w%) and brine, dried ($MgSO_4$), filtered and the filtrate was concentrated. The crude product was purified by chromatography (SiO_2, toluene/EtOAc 1:2 → 0:1) yielding disaccharide **11** (29 mg, 55%) as a colorless oil: R_f 0.31 (toluene/EtOAc 1:4); ^{1}H NMR ($CDCl_3$) = δ 7.38 - 7.29 (m, 5H, Ar), 5.50 (d, 1H, *J* 9.7 Hz, N*H*), 5.40 - 5.38 (m, 1H, H-5'), 5.34 (ddd, 1H, $J_{7',6'}$ 9.4, $J_{7',8'b}$ 4.5, $J_{7',8'a}$ 2.6 Hz, H-7'), 5.05 (dd, 1H, $J_{4',3'}$ 4.8, $J_{4',5'}$ 3.6 Hz, H-4'), 4.71 (d, 1H, *J* 11.8 Hz, C*H*HPh), 4.67 - 4.64 (m, 3H, H-1, H-8'a, CH*H*Ph), 4.73 (b d, 1H, H-3'), 4.42 (dd, 1H, $J_{6'5'}$ 2.0 Hz, H-6'), 4.22 (app td, 1H, $J_{2,3}$ 9.8, $J_{2,1}$ 4.0 Hz, H-2), 4.19 (dd, 1H, $J_{8'b,8'a}$ 12.5 Hz, H-8'b), 3.85 (s, 3H, CO_2CH_3), 3.74 (ddd, 1H, $J_{5,4}$ 9.6, $J_{5,6a}$ 7.1, $J_{5,6b}$ 2.2 Hz, H-5), 3.67 (dd, 1H, $J_{6a,6b}$ 10.0 Hz, H-6a), 3.56 (dd, 1H, H-6b), 3.53 (app t, 1H, $J_{3,4}$ 9.4 Hz, H-3), 3.48 (app dt, 1H, $J_{4,OH}$ 2.7 Hz, H-4) 3.38 (s, 3H, OCH_3), 2.54 (d, 1H, O*H*), 2.12, 2.06, 2.04, 1.98 and 1.92 ppm (5 s, each 3H, COCH_3); ^{13}C NMR ($CDCl_3$) = δ 170.5, 170.2, 169.8, 169.6, 169.3 and 166.1 (6 s, 6C, 5x *C*OCH$_3$, C-1'), 138.1 (s, 1C, Ar), 128.7, 128.11 and 128.08 (3 d, 5C, Ar), 101.3 (s, C-2'), 98.6 (d, C-1), 80.5 (d, C-3), 74.0 (t, *C*H$_2$Ph), 70.7 (d, C-4), 70.1 (d, C-5), 68.3 (d, C-6'), 67.9 (d, C-7'), 65.5 (t, C-6), 65.3 (d, C-4'), 63.4 (d, C-5'), 62.0 (t, C-8'), 55.1 (q, O*C*H$_3$), 53.1 (q, CO_2CH_3), 51.9 (d, C-2), 23.4, 21.9, 20.9, 20.81, 20.75 and 20.6 ppm (5 q, 1 d, 6C, 5 x CO*C*H$_3$, C-3'); LC-MS[S11]: m/z found: 854.45 [M+H$^+$]; 876.45 [M+Na$^+$].

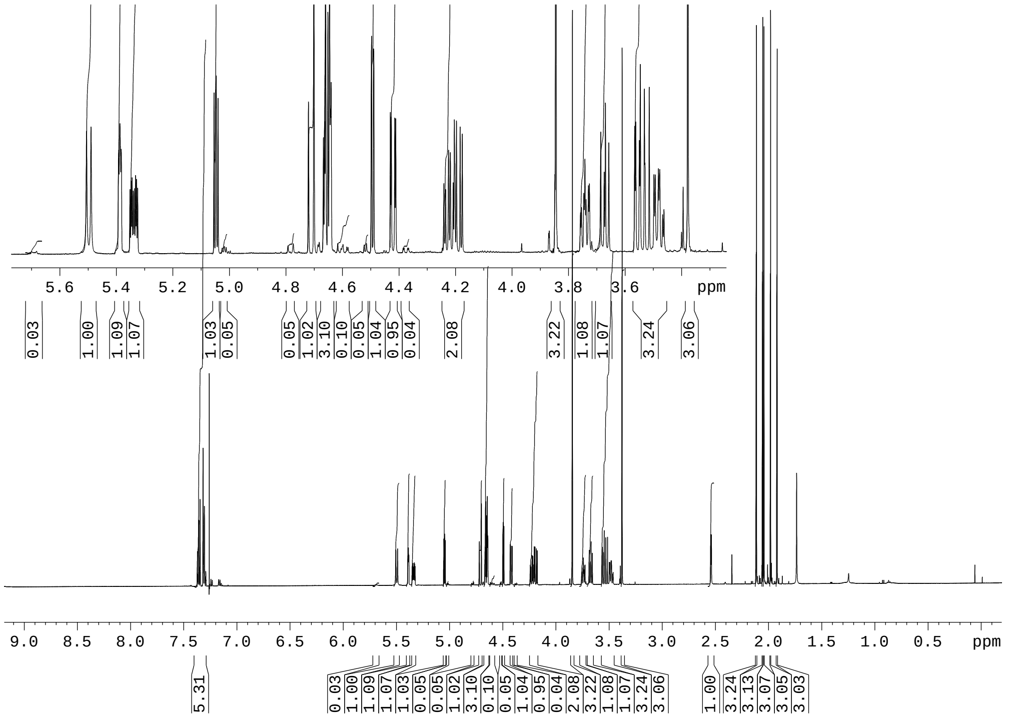

Fig. S1: ^{1}H NMR ($CDCl_3$, 600 MHz) of compound (11).

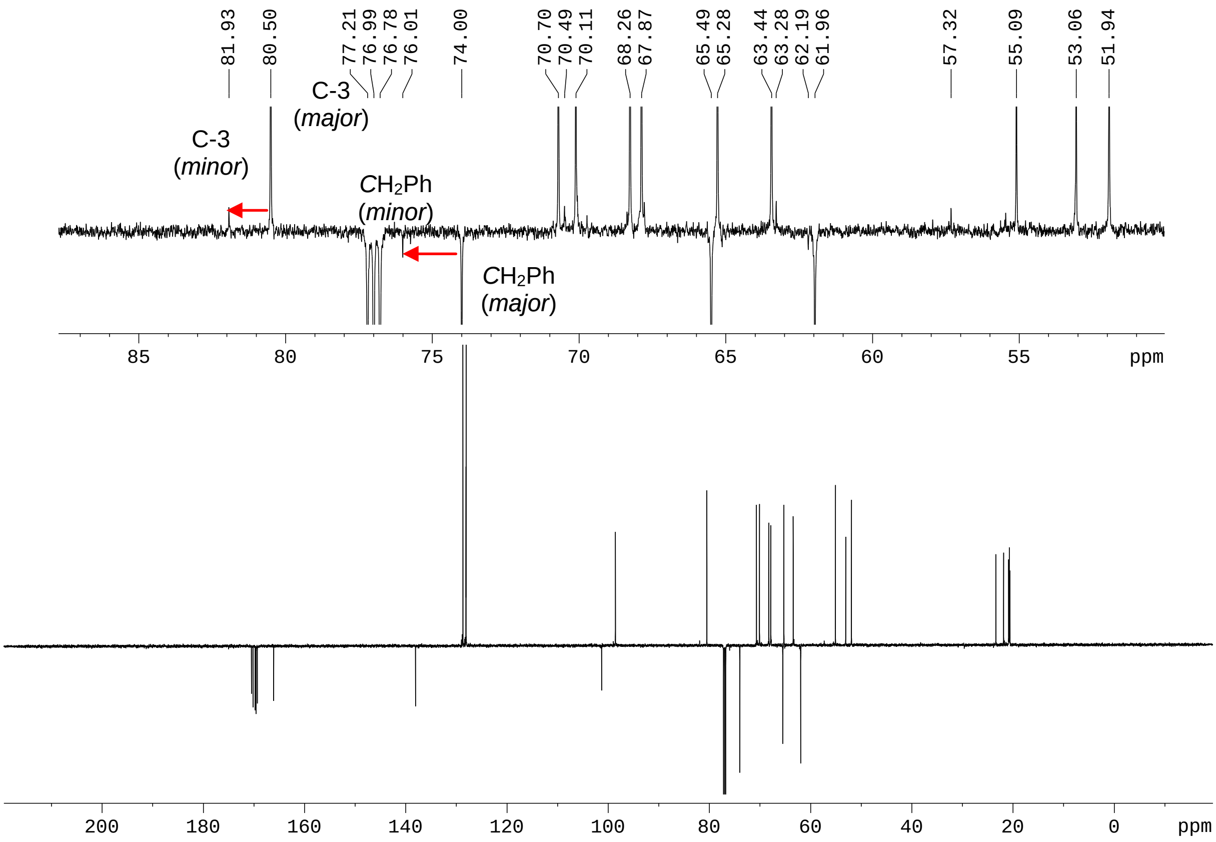

Fig. S2: ^{13}C NMR ($CDCl_3$, 150 MHz) of compound (11).

3.3. Methyl (4,5,7,8-tetra-*O*-acetyl-3-deoxy-3-iodo-D-*glycero*-α-D-*talo*-oct-2-ulopyranosyl)onate-(2→6)-methyl 2-acetamido-4-*O*-acetyl-3-*O*-benzyl-2-deoxy-α-D-glucopyranoside (12)

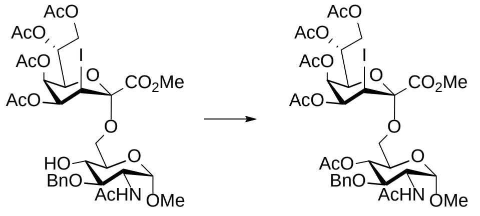

11 **12**

A solution of compound **11** in dry pyridine (3 mL) was treated with 4-(*N,N*-dimethylamino)pyridine (1 mg) and acetic anhydride (0.3 mL) for 5 h at room temperature. The mixture was cooled (0 °C) and dry MeOH (1 mL) was added slowly. After 5 min the mixture was coevaporated with toluene (3 x) and the residue was purified by chromatography (toluene/EtOAc 1:3) followed by HPLC (toluene/EtOAc 1:2) which afforded disaccharide **12** (13 mg, 59%) as a colorless oil: R_f 0.30 (CH_2Cl_2/EtOAc 1:1, HP-TLC); ^{1}H NMR ($CDCl_3$) = δ 7.35 - 7.23 (m, 5H, Ar), 5.39 - 5.37 (m, 1H, 5'), 5.33 - 5.30 (m, 2H, H-7', N*H*), 5.08 (dd, 1H, $J_{4',3'}$ 4.7, $J_{4',5'}$ 3.7 Hz, H-4'), 4.90 (dd, 1H, $J_{4,3}$ 10.5, $J_{4,5}$ 9.2 Hz, H-4), 4.74 - 4.70 (m, 2H, H-1, H-8'a), 4.62 (d, 1H, *J* 11.6 Hz, C*H*HPh), 4.52 - 4.49 (m, 2H, H-3', CH*H*Ph), 4.37 (dd, 1H, $J_{6',7'}$ 9.4, $J_{6',5'}$ 2.3 Hz, H-6'), 4.26 (ddd, 1H, $J_{2,NH}$ 10.6, $J_{2,3}$ 9.1, $J_{2,1}$ 3.6 Hz, H-2), 4.15 (dd, 1H, $J_{8'b,8'a}$ 12.6, $J_{8'b,7'}$ 4.2 Hz, H-8'b), 3.86 (ddd, 1H, $J_{5,6a}$ 8.2, $J_{5,6b}$ 1.7 Hz, H-5), 3.84 (s, 3H, CO_2CH_3), 3.71 (dd, 1H, H-3), 3.64 (dd, 1H, $J_{6a,6b}$ 10.4 Hz, H-6a), 3.40 (s, 3H, OCH_3), 3.25 (dd, 1H, H-6b), 2.12, 2.11, 2.04, 2.02, 1.99 and 1.87 ppm (6 s, each 3H, $COCH_3$); ^{13}C NMR ($CDCl_3$) = δ 170.4, 170.1, 169.7, 169.6, 169.5, 169.3 and 166.1 (7 s, 7C, 6x *C*OCH$_3$, C-1'), 137.8 (s, 1C, Ar), 128.5, 128.1 and 128.0 (3 d, 5C, Ar), 101.3 (s, C-2'), 98.2 (d, C-1), 77.5 (d, C-3), 73.1 (t, *C*H$_2$Ph), 70.5 (d, C-4), 68.9 (d, C-5), 68.4 (d, C-6'), 67.9 (d, C-

7’), 65.2 (d, C-4’), 64.9 (t, C-6), 63.5 (d, C-5’), 61.8 (t, C-8’), 55.3 (d, OCH_3), 52.9 (q, CO_2CH_3), 51.9 (d, C-2), 23.3, 22.0, 20.89, 20.86, 20.8, 20.7 and 20.6 ppm (6 q, 1 d, 7C, 6 x COCH_3, C-3’); LC-MS[S11]: m/z found: 896.50 [M+H^+]; 918.50 [M+Na^+].

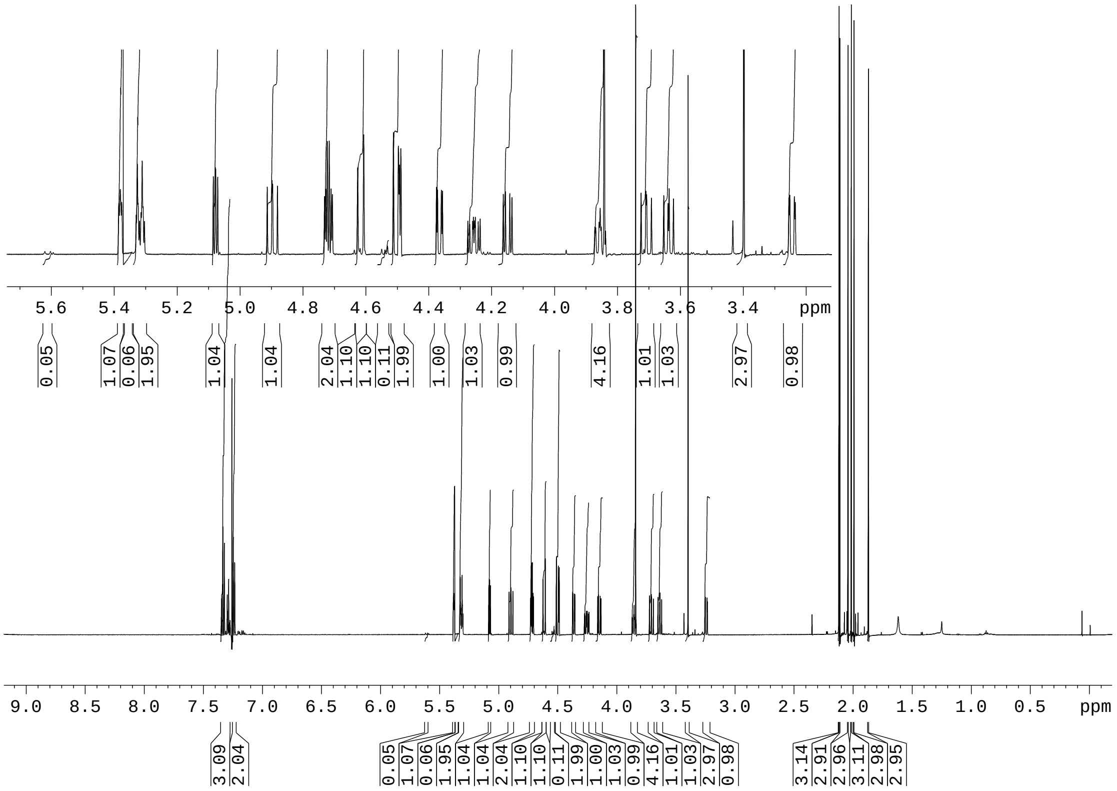

Fig. S3: ^{1}H NMR ($CDCl_3$, 600 MHz) of compound (12).

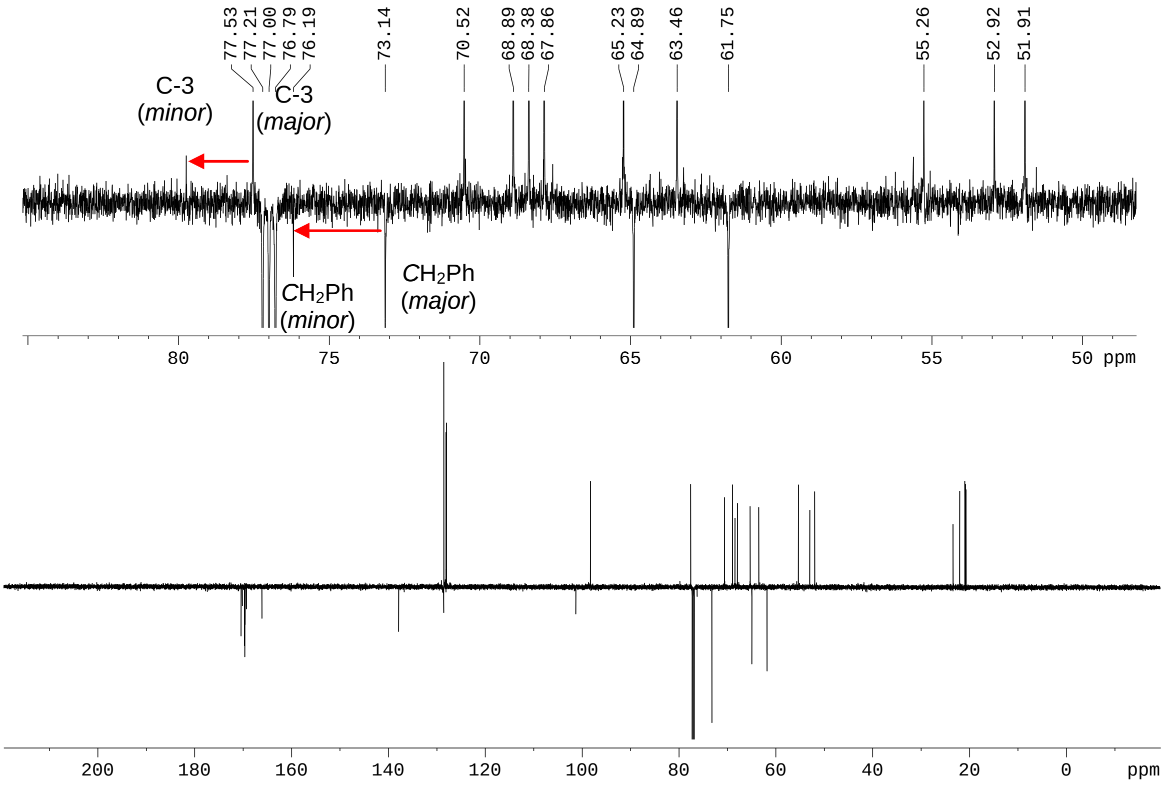

Fig. S4: ^{13}C NMR ($CDCl_3$, 150 MHz) of compound (12).

3.4. Methyl (4,5,7,8-tetra-*O*-acetyl-3-deoxy-α-D-*manno*-oct-2-ulopyranosyl)onate-(2→6)-methyl 2-acetamido-4-*O*-acetyl-3-*O*-benzyl-2-deoxy-α-D-glucopyranoside (13)

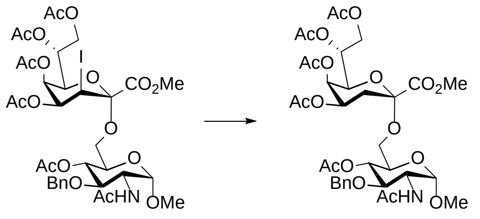

12 **13**

Compound **12** (12.0 mg, 0.013 mmol) was suspended in dry cyclohexane (4.0 mL) and dry 1,2-dichloroethane (0.5 mL). After degassing with argon the mixture was refluxed for 15 min followed by addition of lauroyl peroxide (1.9 mg, 0.005 mmol). Refluxing for 2 h and solvent evaporation afforded a crude product which was separated by chromatography (toluene/EtOAc 1:4 → 0.1) and HPLC (EtOAc) providing disaccharide **13** (7.7 mg, 75%) as a colorless oil: R_f 0.28 (CH_2Cl_2/EtOAc 1:1, HP-TLC); ^{1}H NMR ($CDCl_3$) = δ 7.35 - 7.24 (m, 5H, Ar), 5.38 - 5.31 (m, 3H, N*H*, H-4', H-5'), 5.19 (ddd, 1H, $J_{7',6'}$ 9.3, $J_{7',8'b}$ 4.7, $J_{7',8'a}$ 2.4 Hz, H-7'), 4.91 (dd, 1H, $J_{4,5}$ 10.4, $J_{4,3}$ 9.1 Hz, H-4), 4.72 (d, 1H, $J_{1,2}$ 3.8 Hz, H-1), 4.65 (dd, 1H, $J_{8'a,8'b}$ 12.4 Hz, H-8'a), 4.63 (d, 1H, *J* 11.7 Hz, C*H*HPh), 4.51 (d, 1H, *J* 11.6 Hz, CH*H*Ph), 4.29 - 4.24 (m, 2H, H-2, H-6'), 4.06 (dd, 1H, H-8'b), 3.88 (ddd, 1H, $J_{5,6a}$ 8.2, $J_{5,6b}$ 2.1 Hz, H-5), 3.80 (s, 3H, CO_2CH_3), 3.72 (dd, 1H, $J_{3,2}$ 10.7 Hz, H-3), 3.57 (dd, 1H, $J_{6a,6b}$ 10.6 Hz, H-6a), 3.49 (dd, 1H, H-6b), 3.41 (s, 3H, OCH_3), 2.13 - 2.07 (m, 8H, H-3'*eq*, H-3'*ax*, 2 x COCH_3), 2.03, 2.02, 1.96 and 1.88 ppm (4 s, each 3H, COCH_3); ^{13}C NMR ($CDCl_3$) = δ 170.5, 170.4, 169.83, 169.82, 169.7, 169.6 and 167.4 (7 s, 7C, 6 x *C*OCH_3, C-1'), 137.9 (s, 1C, Ar), 128.5, 128.1 and 127.9 (3 d, 5C, Ar), 98.4 (s, C-2'), 98.2 (d, C-1), 77.6 (d, C-3), 73.1 (t, *C*H_2Ph), 70.8 (d, C-4), 69.1 (d, C-5), 68.8 (d, C-6'), 68.0 (d, C-7'), 66.2 (d, C-4'), 64.6 (d, C-5'), 63.2 (t, C-6), 62.0 (t, C-8'), 55.3 (q, OCH_3), 52.7 (q, CO_2CH_3), 51.9 (d, C-2), 32.0 (t, C-3'), 23.4, 20.9, 20.77, 20.75, 20.72 and 20.66 ppm (6 q, 6C, COCH_3); LC-MS[S11]: m/z found: 770.55 [M+H$^+$]; 792.55 [M+Na$^+$].

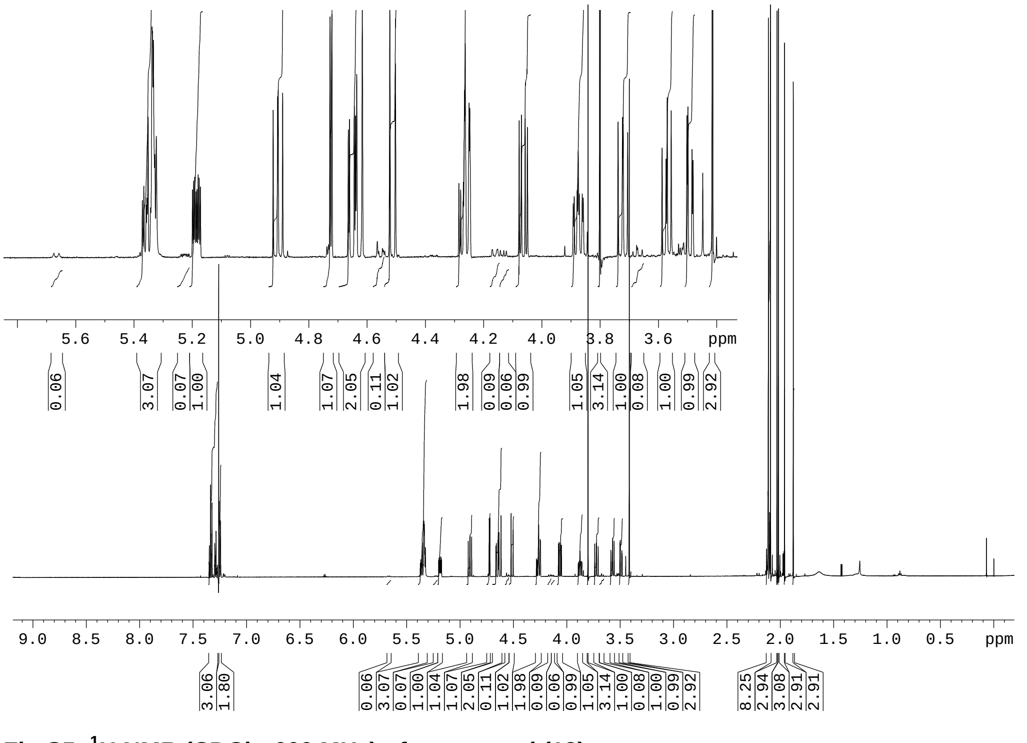

Fig S5: ^{1}H NMR ($CDCl_3$, 600 MHz) of compound (13).

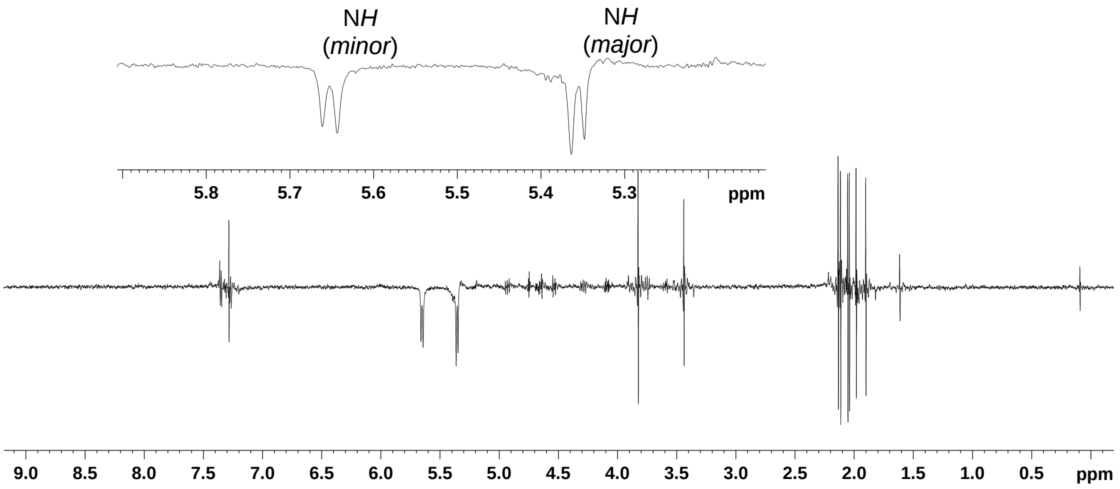

Fig S6: 1D-NOE-difference spectrum ($CDCl_3$, 600 MHz) of compound (13); selective pulse @ 5.64 ppm (N*H* of minor rotamer).

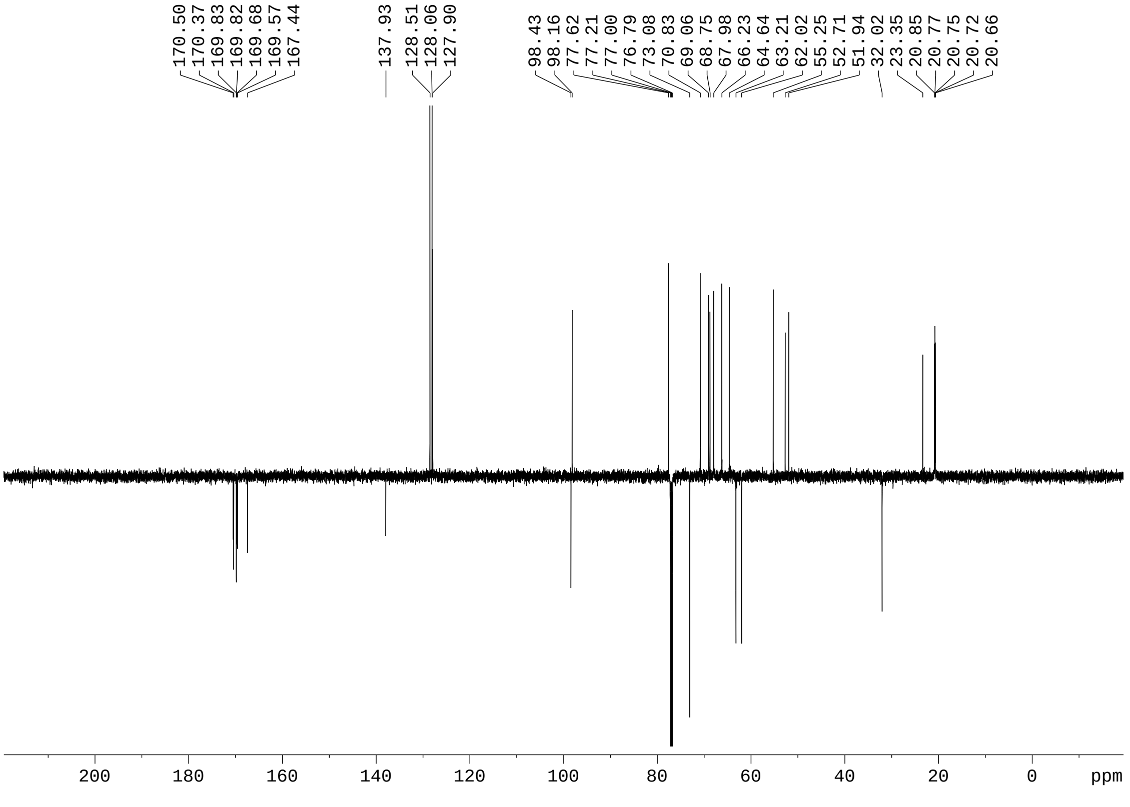

Fig S7: ^{13}C NMR ($CDCl_3$, 150 MHz) of compound (13).

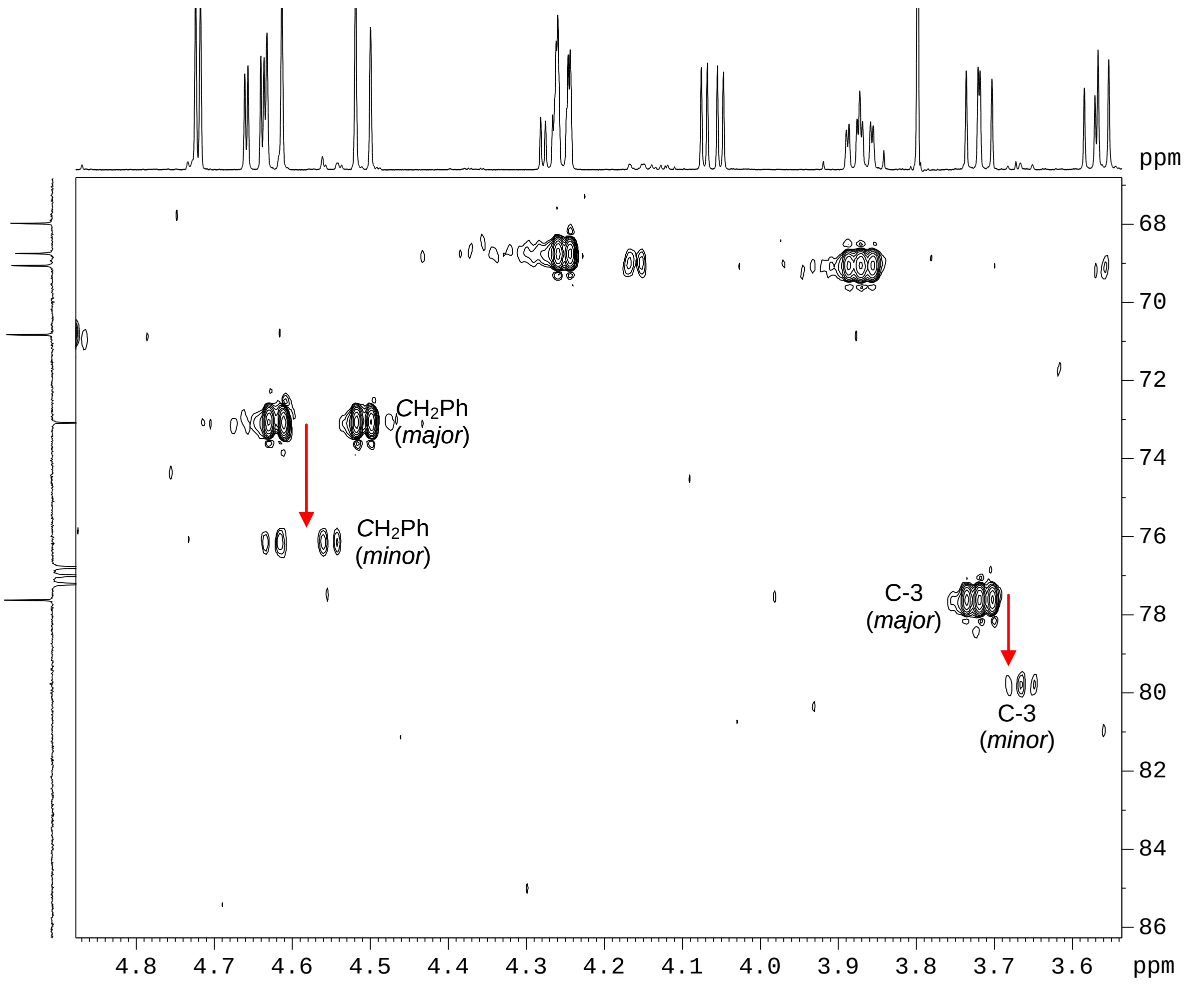

Fig S8: HSQC spectrum ($CDCl_3$, 600 MHz) of compound (13).

4. NMR studies on competitive iodonium ion migration

4.1. Experimental procedure

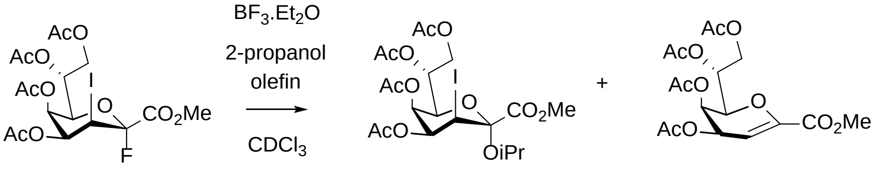

1 **9** **6**

glycosylation pathway *iodonium-migration pathway*

The 3-iodo donor **1** (10 mg, 0.018 mmol) was dissolved in dry $CDCl_3$ (kept over 4 Å molecular sieves overnight) in an NMR tube and 2-propanol (2.8 µL, 0.036 mmol) and the respective olefin (~ 2 eq., see Table 1) were added. From this solution a ^{1}H NMR spectrum was recorded as a reference. Then, the mixture was treated with $BF_3{\cdot}Et_2O$ (7.5 µL, 0.036 mmol), agitated and a ^{1}H spectrum was recorded

immediately. In the presence of 1-octene (entry I) and cyclohexene (entry II) complete consumption of donor **1** was already detected in the first spectrum (reaction time: < 1min). For allyl methyl ether (entry III) ^{1}H spectra were collected over a period of 1 h with variable time intervals in between. The integrals of representative signals for donor **1**, glycal ester **6** and glycoside **9** were collected in relation to the residual solvent peak. The integral of the donor signal in the reference spectrum was defined as 100%, the percentages of the collected integral values were calculated and plotted over time in a diagram (see Fig. S14). For methyl crotonate (entry IV) no effect of the olefin on the glycosylation was observed.

Table S1. Reaction conditions and changes of NMR-integration values

Entry	Olefin	Ratio 6:9	Time	Eq. olefin	Δ-Integral Olefin	Integral Glycal
I	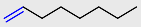	0 : 1	< 1 min	2.3	-0.94	0.92
II	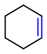	0 : 1	< 1 min	3.0	-0.92	1.02
III	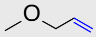	1 : 1.2	60 min	1.5	-0.52	0.46
IV	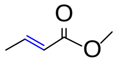	1 : 0	60 min	2.7	+0.05	0.00

To perform a negative control, the same procedure was repeated in the absence of donor **1** (for entries I to III). The ^{1}H-NMR spectra were collected before and after $BF_3.Et_2O$ addition (see below). In all cases the signals of 2-propanol were significantly shifted, the olefin signals remained unchanged or were slightly shifted, respectively. However, no degradation or consumption of the respective olefin was observed.

4.2. 1-Octene (entry I)

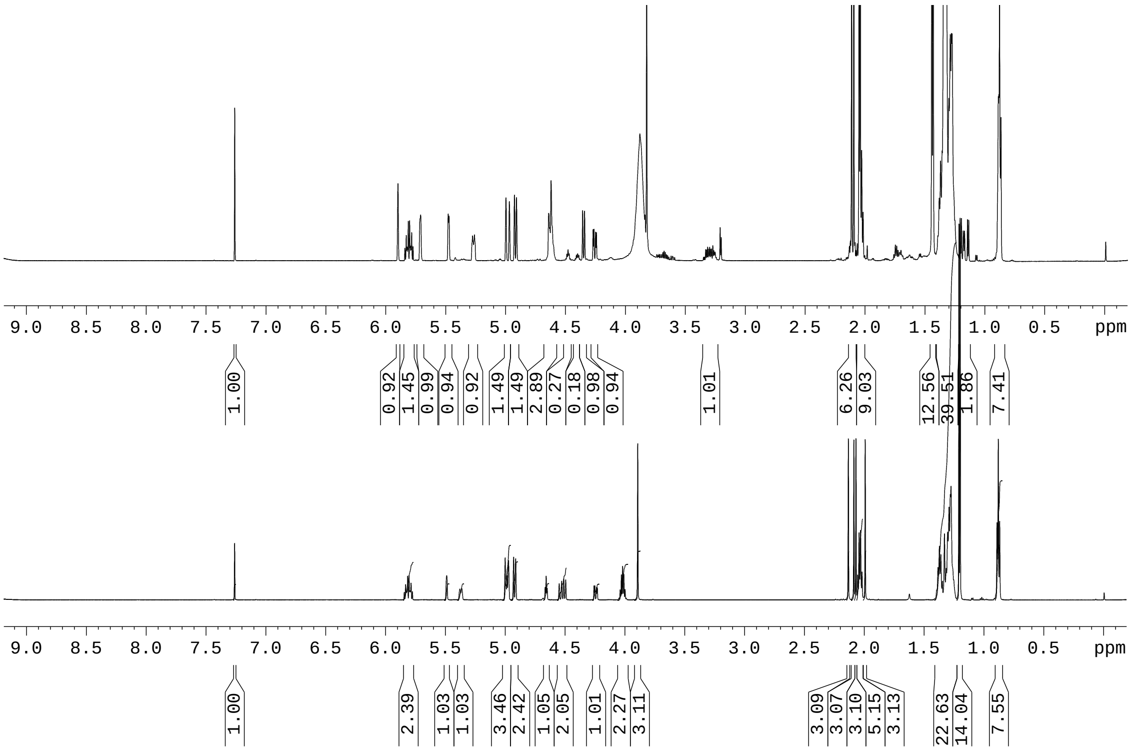

Fig. S9: Comparison of ^{1}H NMR spectra before (bottom) and after (top) donor activation (reaction time < 1 min).

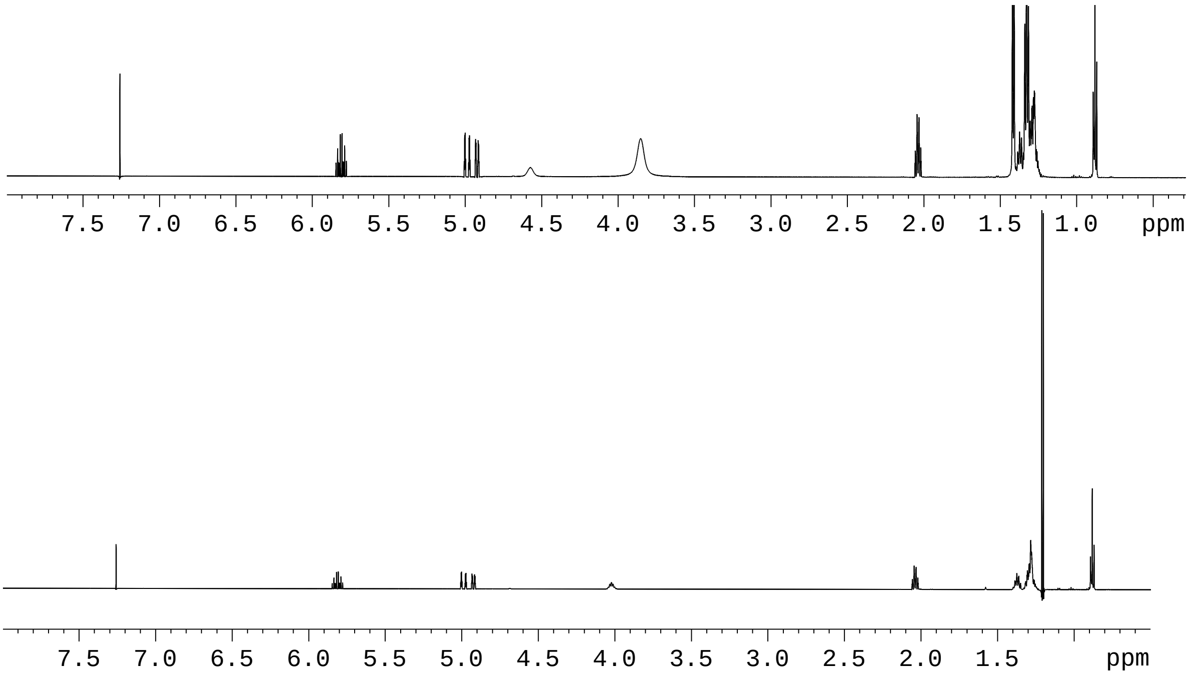

Fig. S10: Negative control: Comparison of a solution without donor (1) before (bottom) and after (top) promotor addition.

4.3. Cyclohexene (entry II)

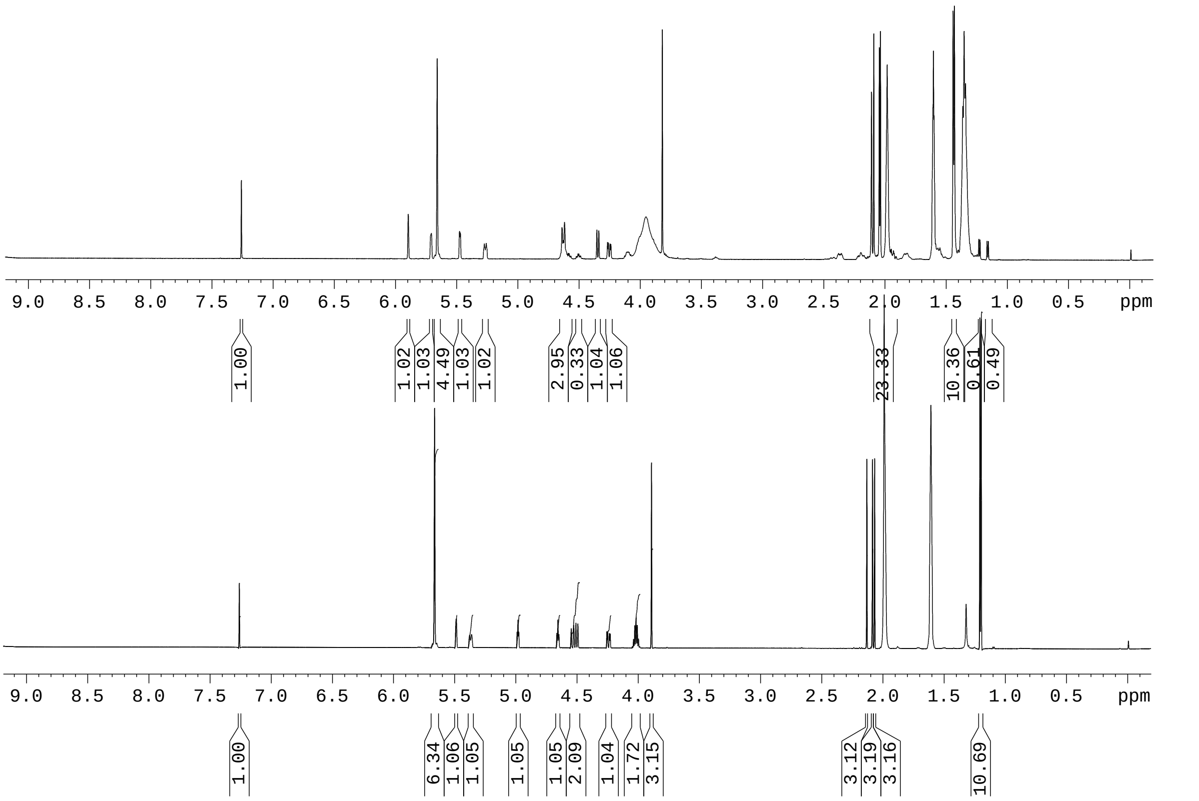

Fig. S11: Comparison of ^{1}H NMR spectra before (bottom) and after (top) donor activation (reaction time < 1 min).

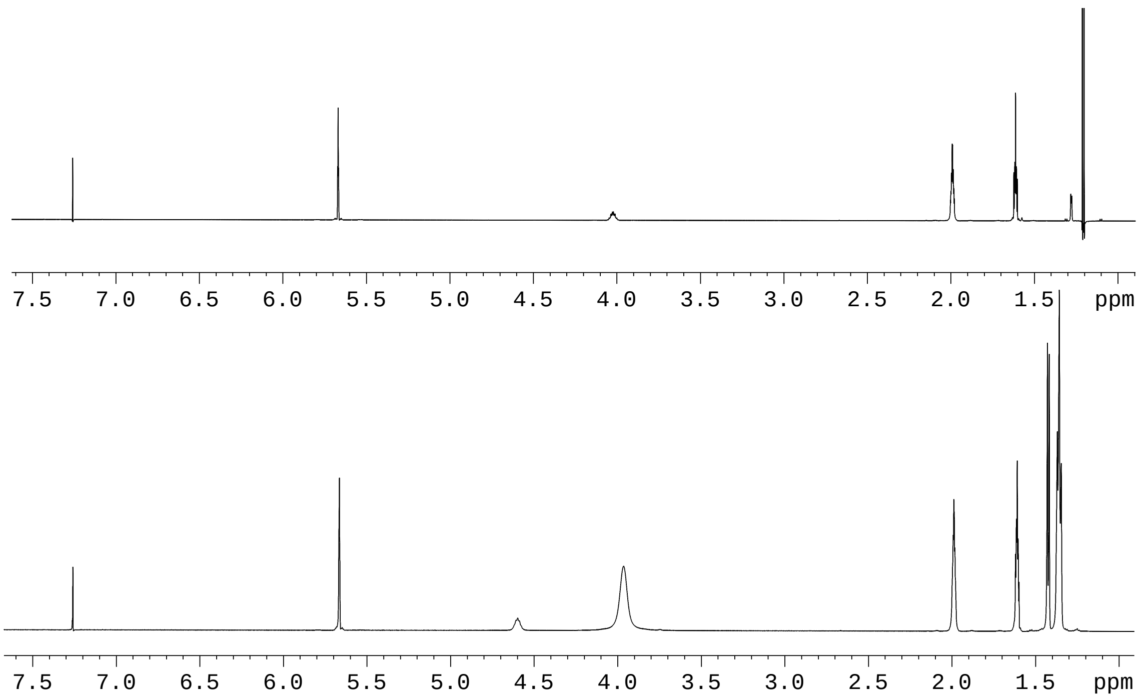

Fig. S12: Negative control: Comparison of a solution without donor (1) before (bottom) and after (top) promotor addition.

4.4. Allyl methyl ether (entry III)

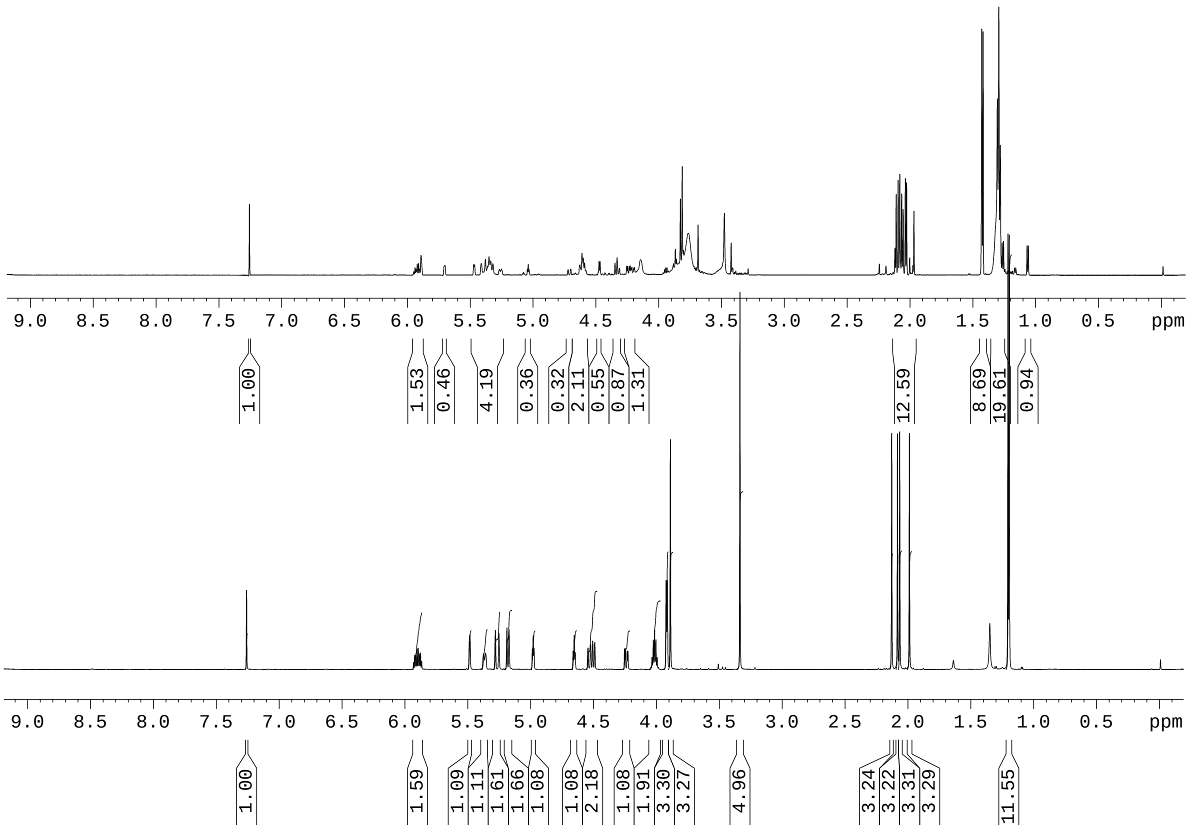

Fig. S13: Comparison of ^{1}H NMR spectra before (bottom) and after (top) donor activation (reaction time = 1h).

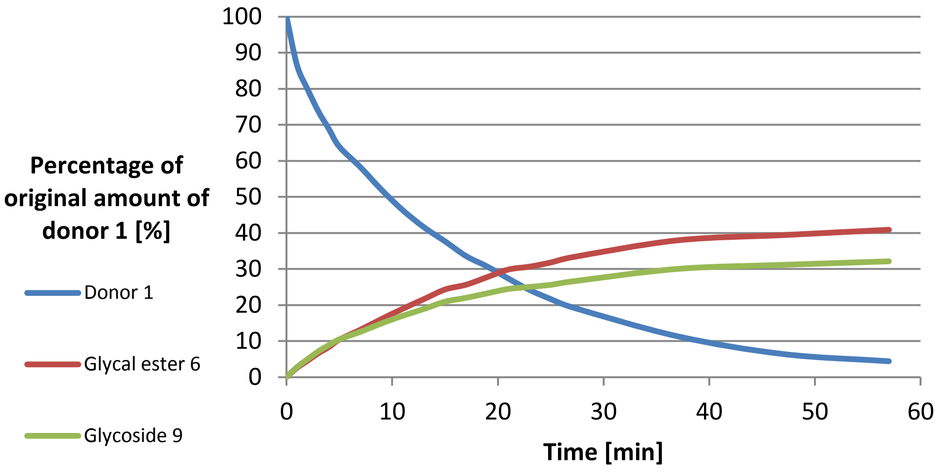

Fig. S14: The decrease of donor **1** correlates with the parallel formation of glycal ester **6** and glycoside **9**. This shows, that both pathways are active. The total amount of glycal **6** and glycoside **9** did not reach 100% due to some residual donor (~ 4%) and formation of hydrolysed donor.

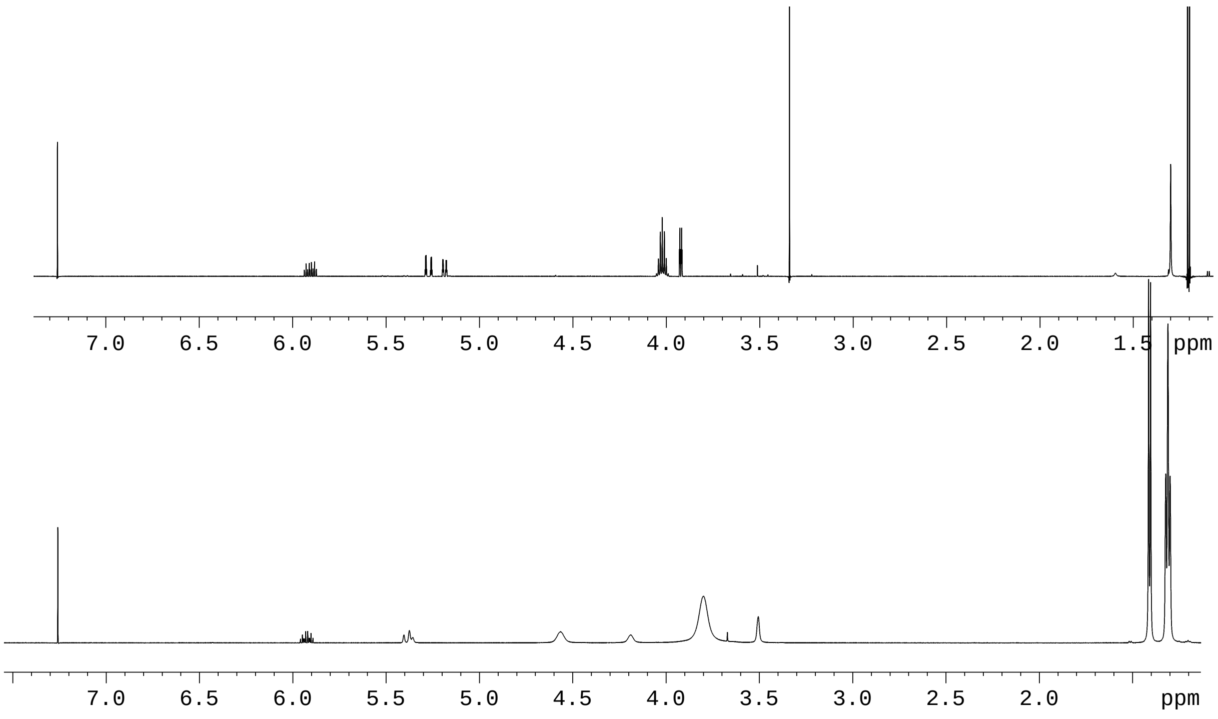

Fig. S15: Negative control: Comparison of a solution without donor 1 before (bottom) and after (top) promotor addition.

4.5. Methyl crotonate (entry IV)

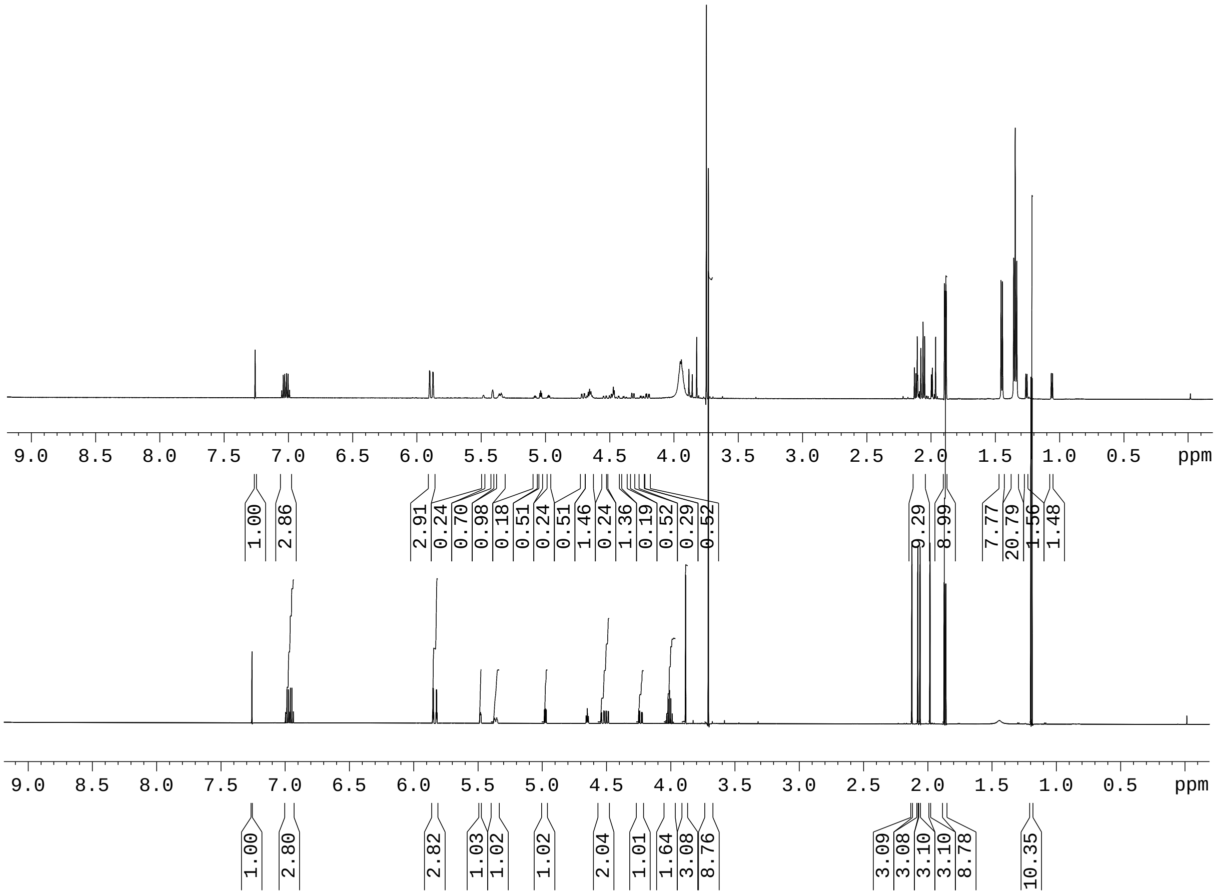

Fig. S16: Comparison of ^{1}H NMR spectra before (bottom) and after (top) donor activation (reaction time = 1 h).

5. NMR spectra of compounds (3) – (5) and (15) – (20)

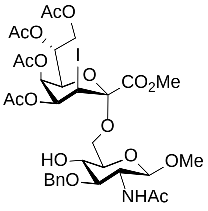

3

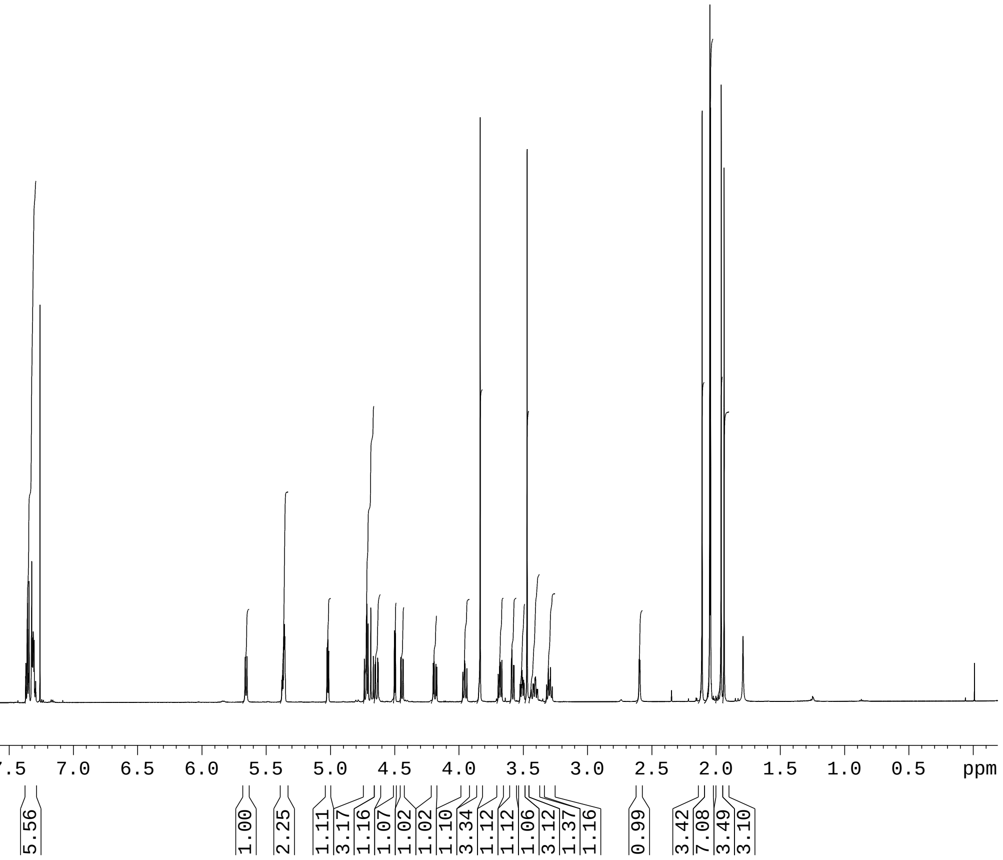

^{1}H NMR ($CDCl_3$, 600 MHz)

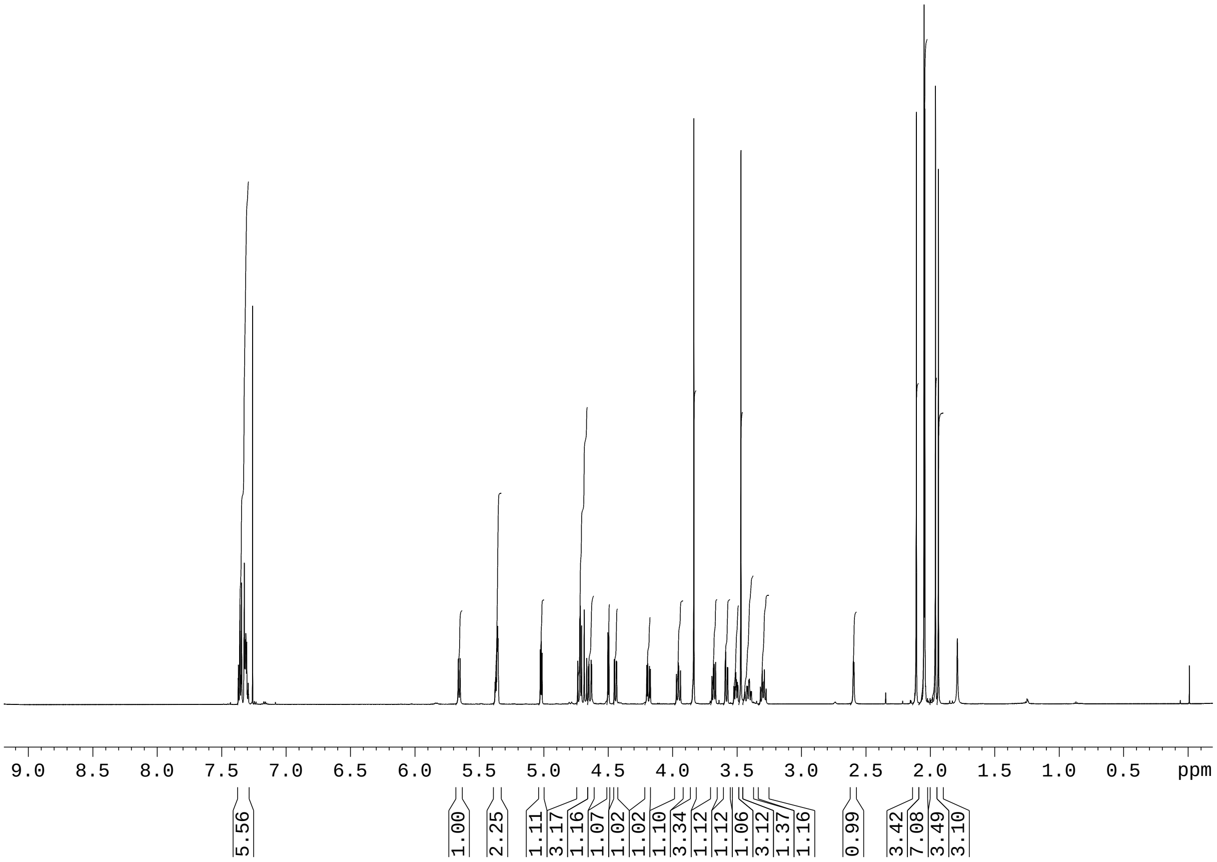

^{13}C NMR ($CDCl_3$, 150 MHz)

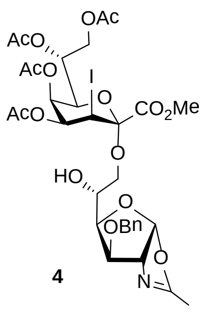

4

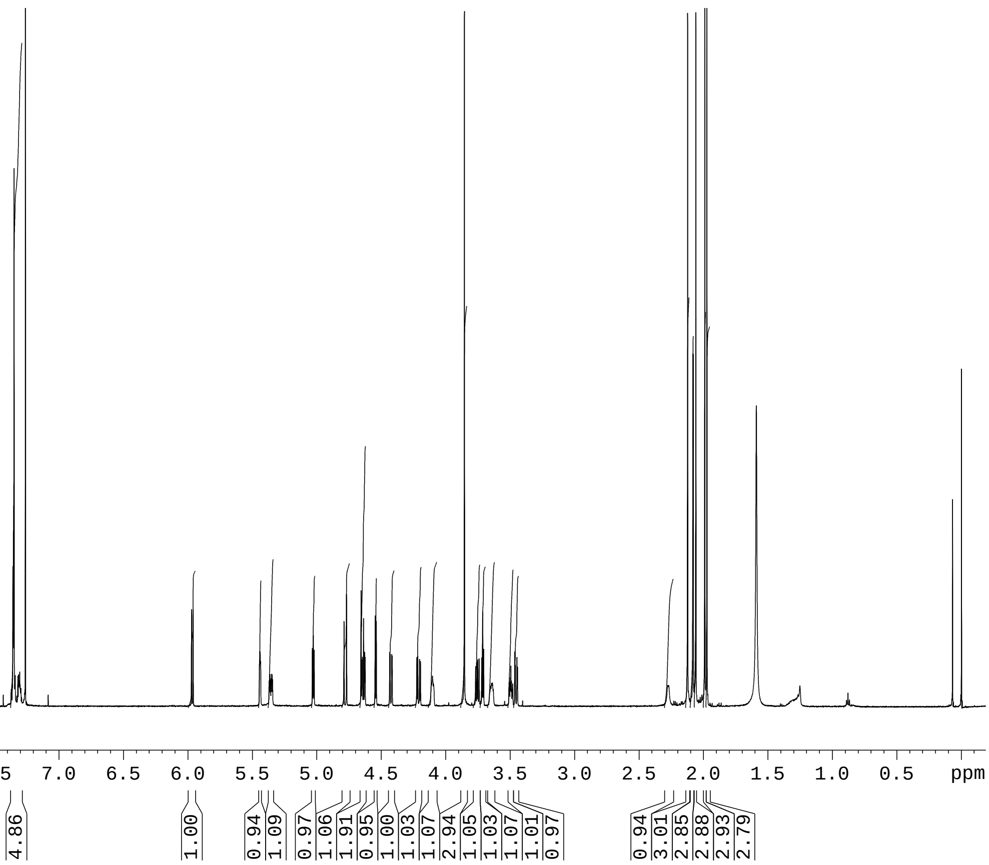

^{1}H NMR ($CDCl_3$, 600 MHz)

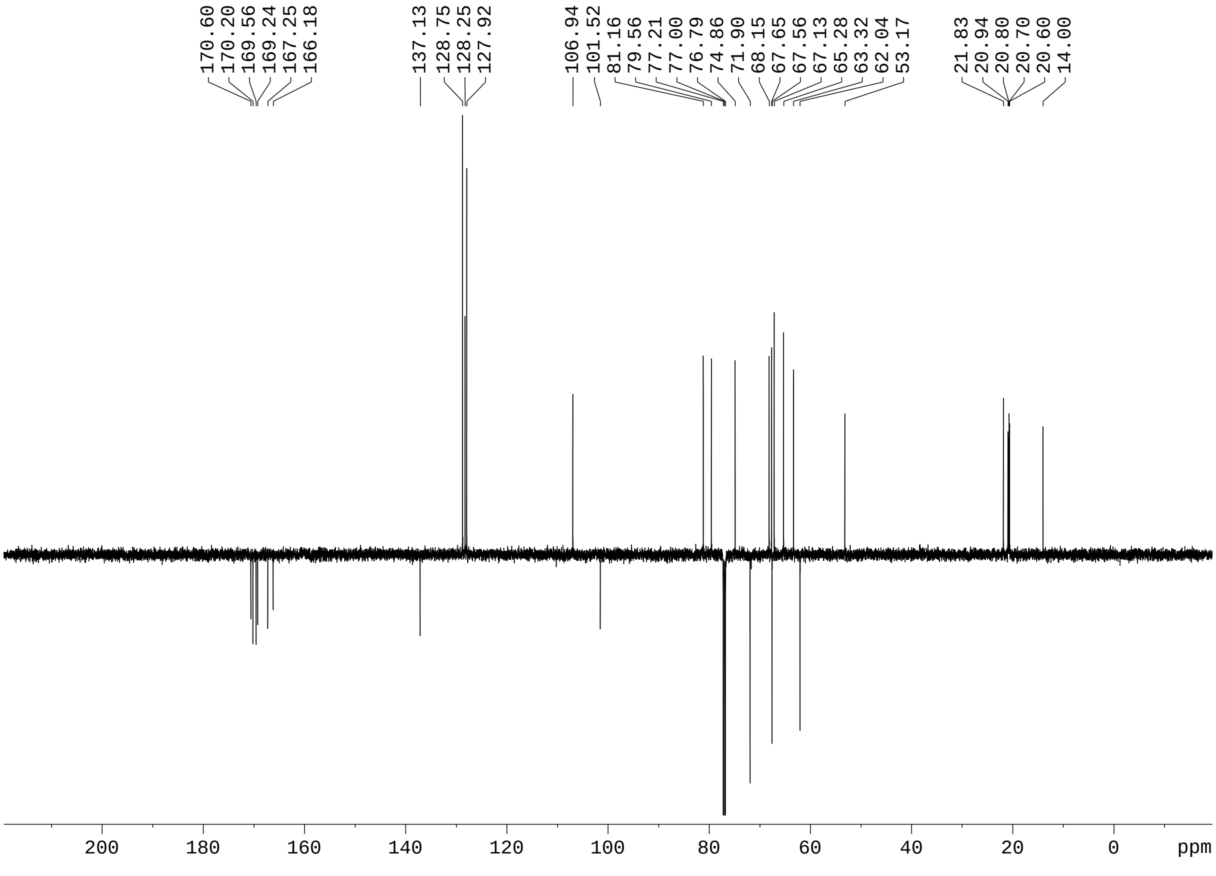

^{13}C NMR ($CDCl_3$, 150 MHz)

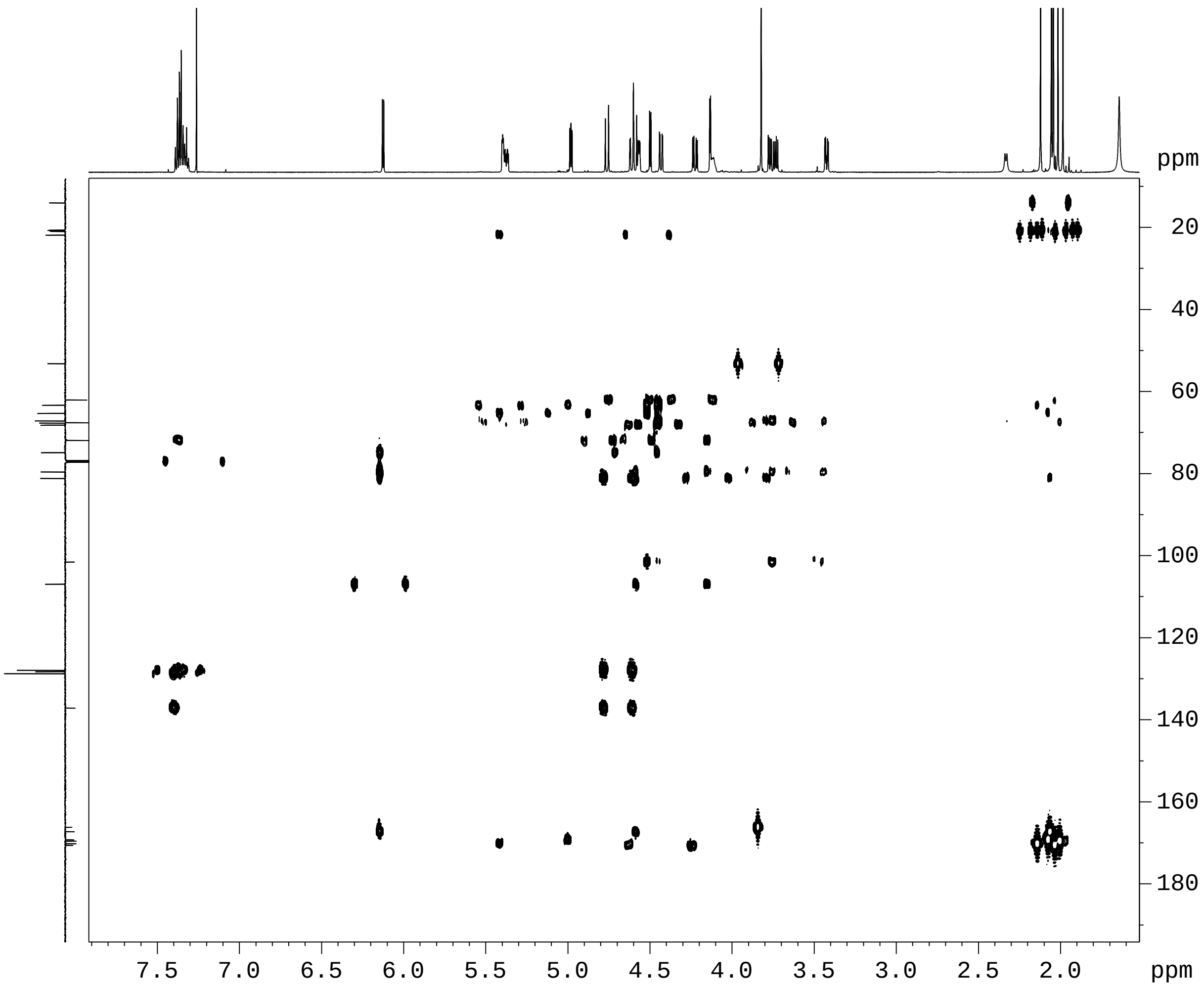

HMBC ($CDCl_3$, 600 MHz)

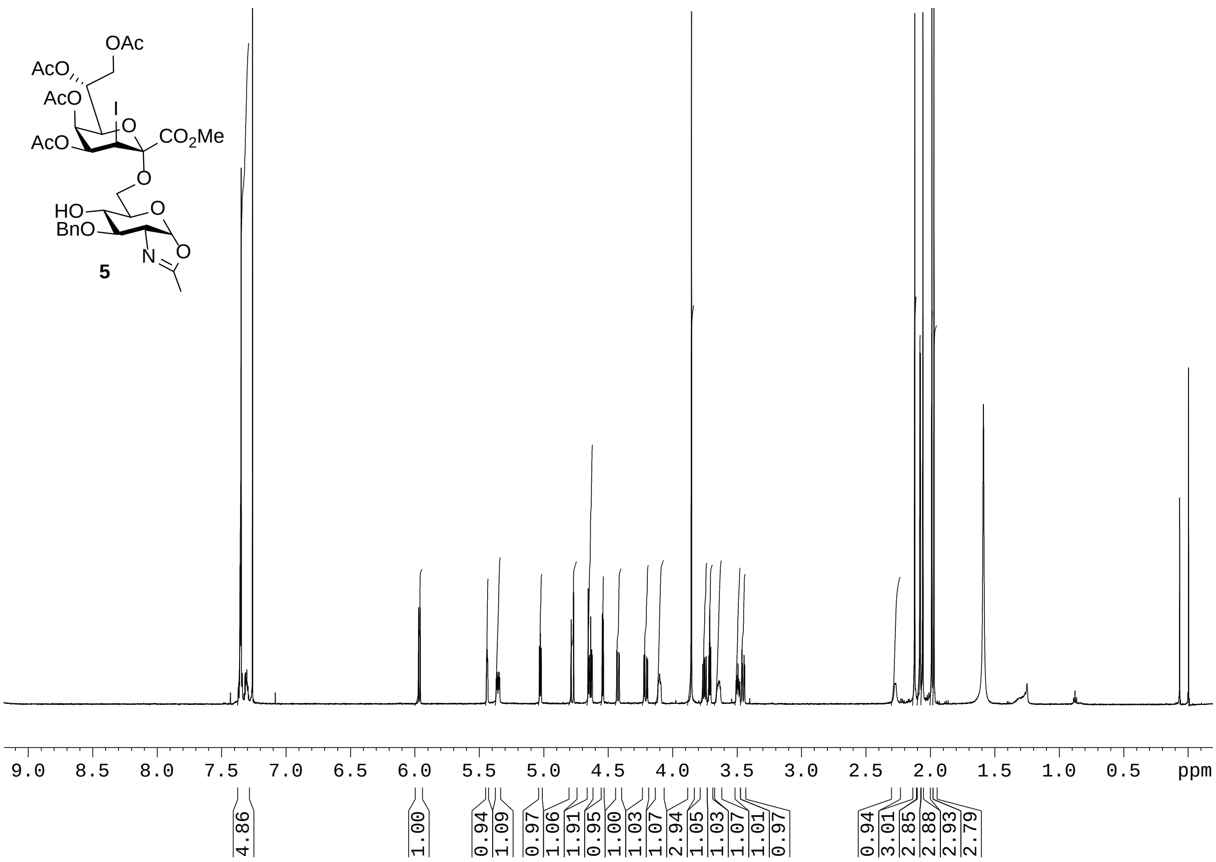

^{1}H NMR ($CDCl_3$, 600 MHz)

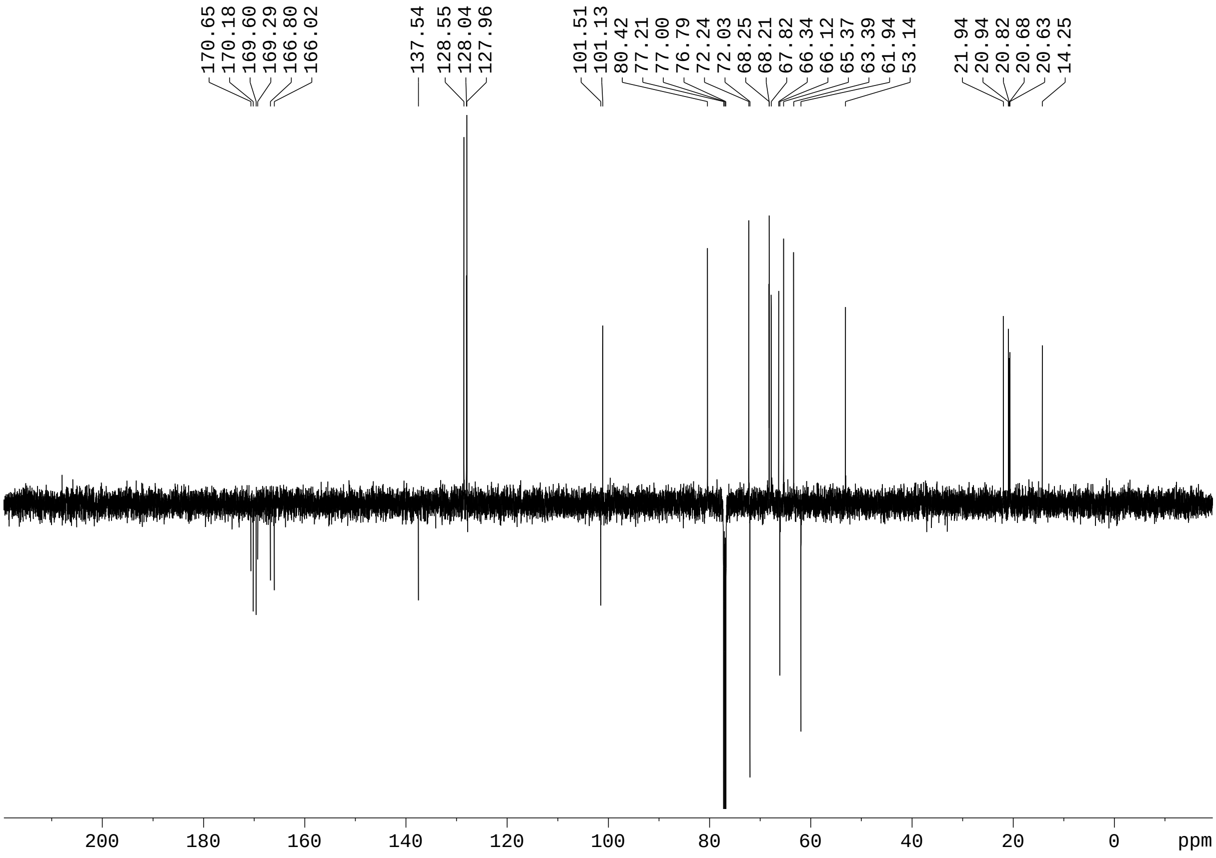

^{13}C NMR ($CDCl_3$, 150 MHz)

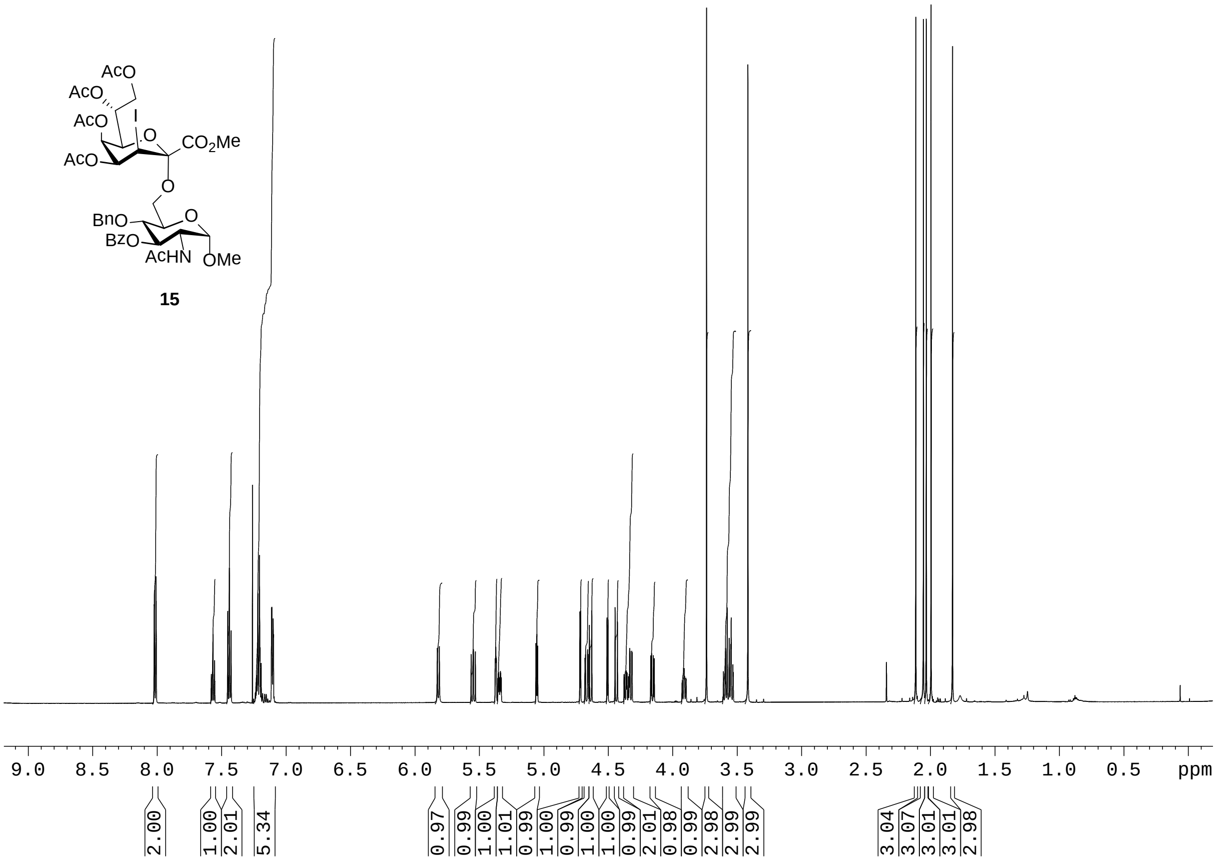

^{1}H NMR ($CDCl_3$, 600 MHz)

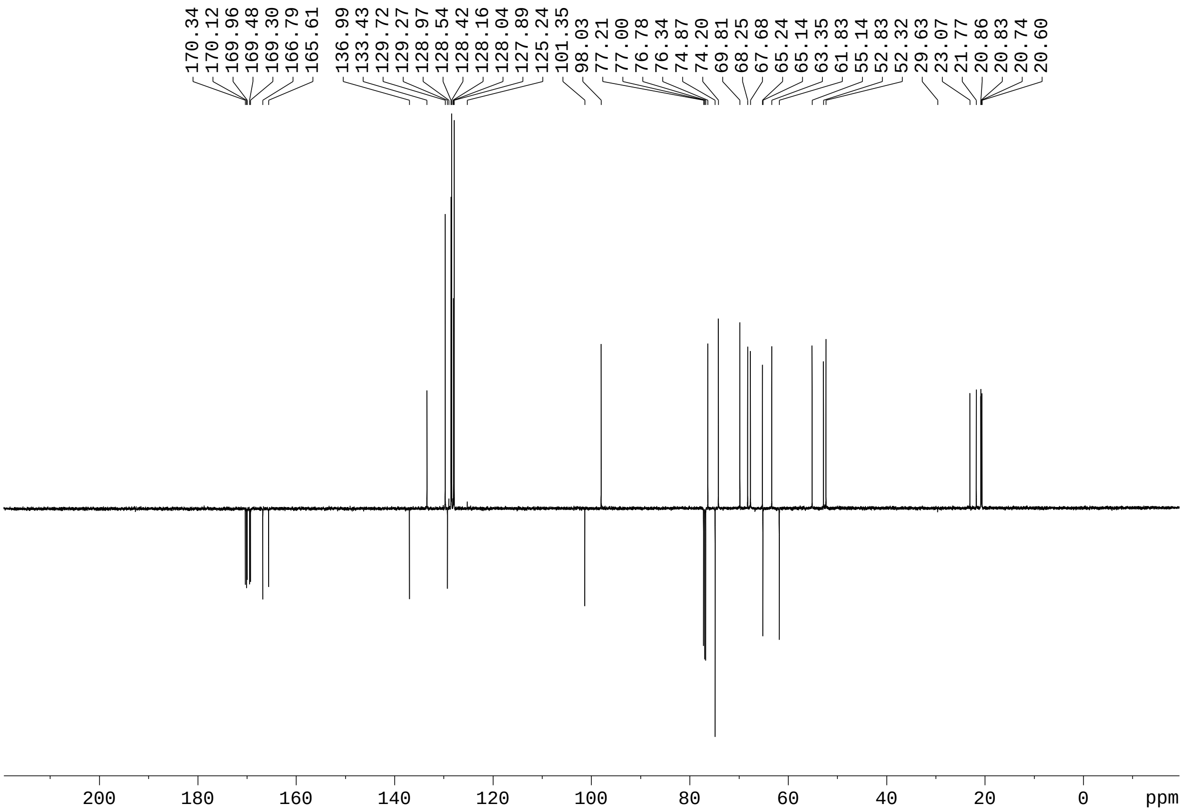

^{13}C NMR ($CDCl_3$, 150 MHz)

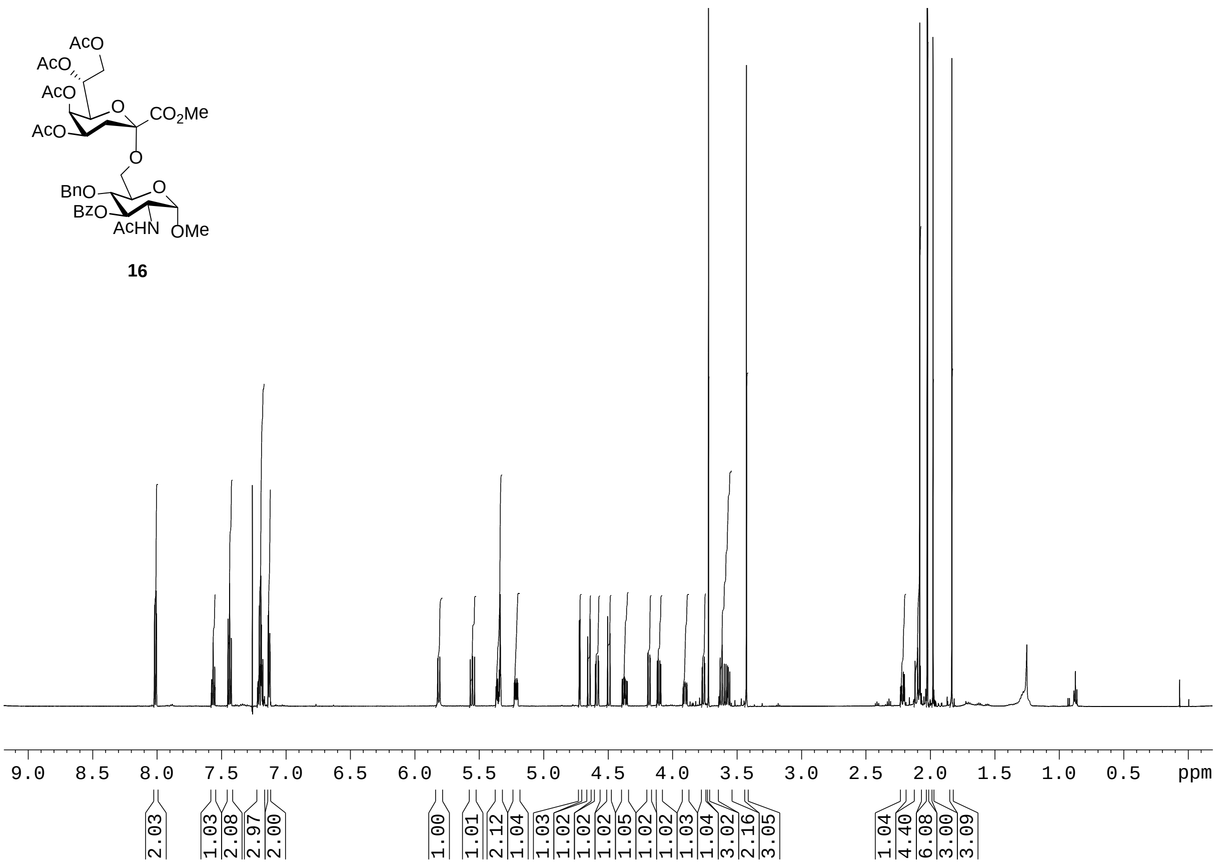

^{1}H NMR (CDCl$_3$, 600 MHz)

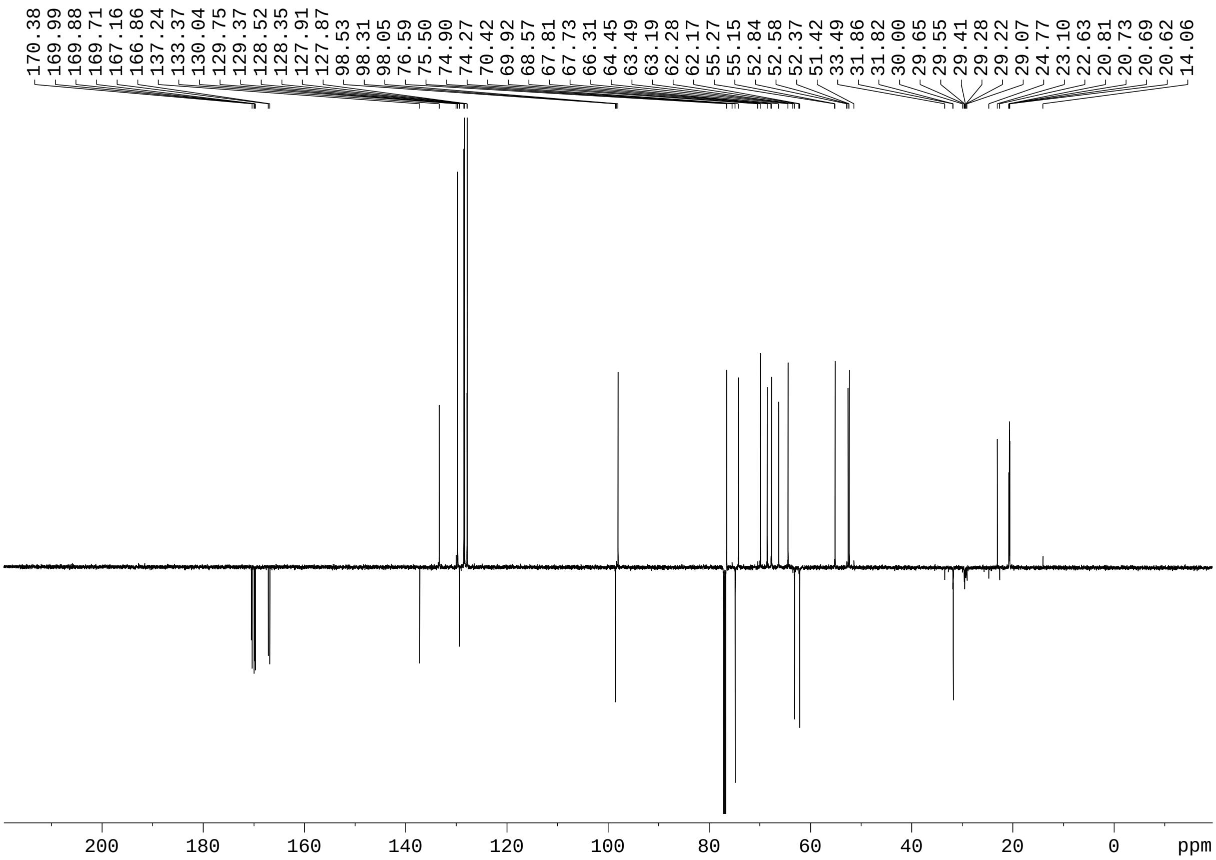

^{13}C NMR (CDCl$_3$, 150 MHz)

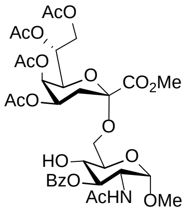

17

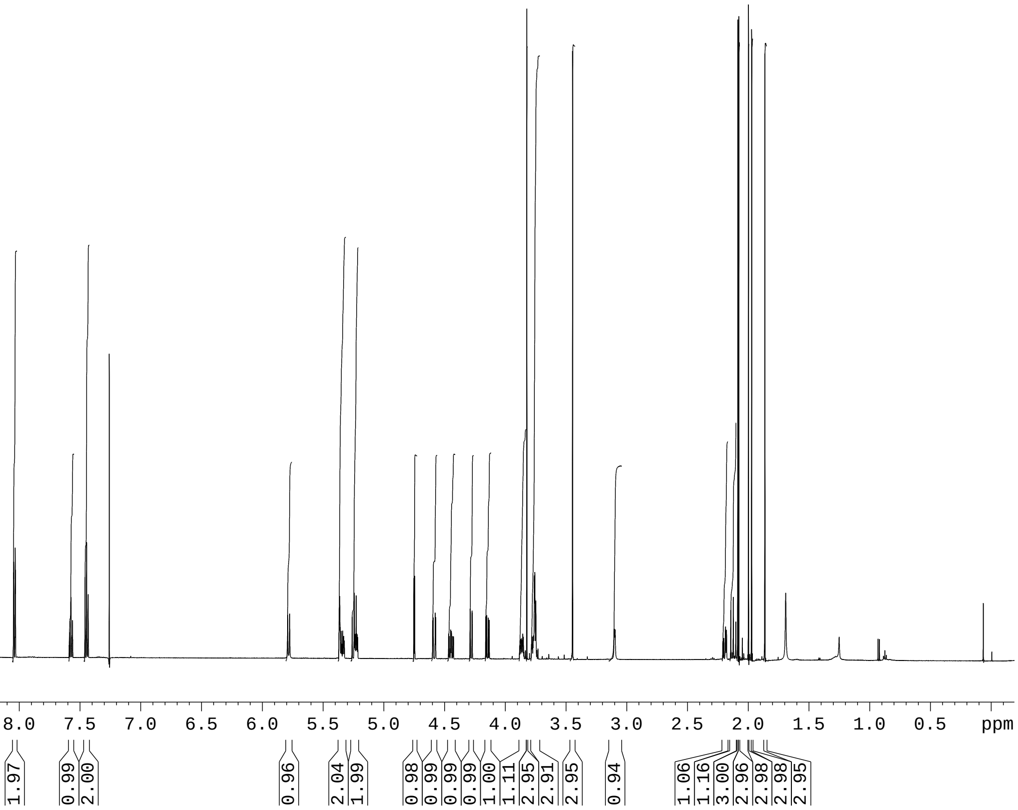

^{1}H NMR (CDCl$_3$, 600 MHz)

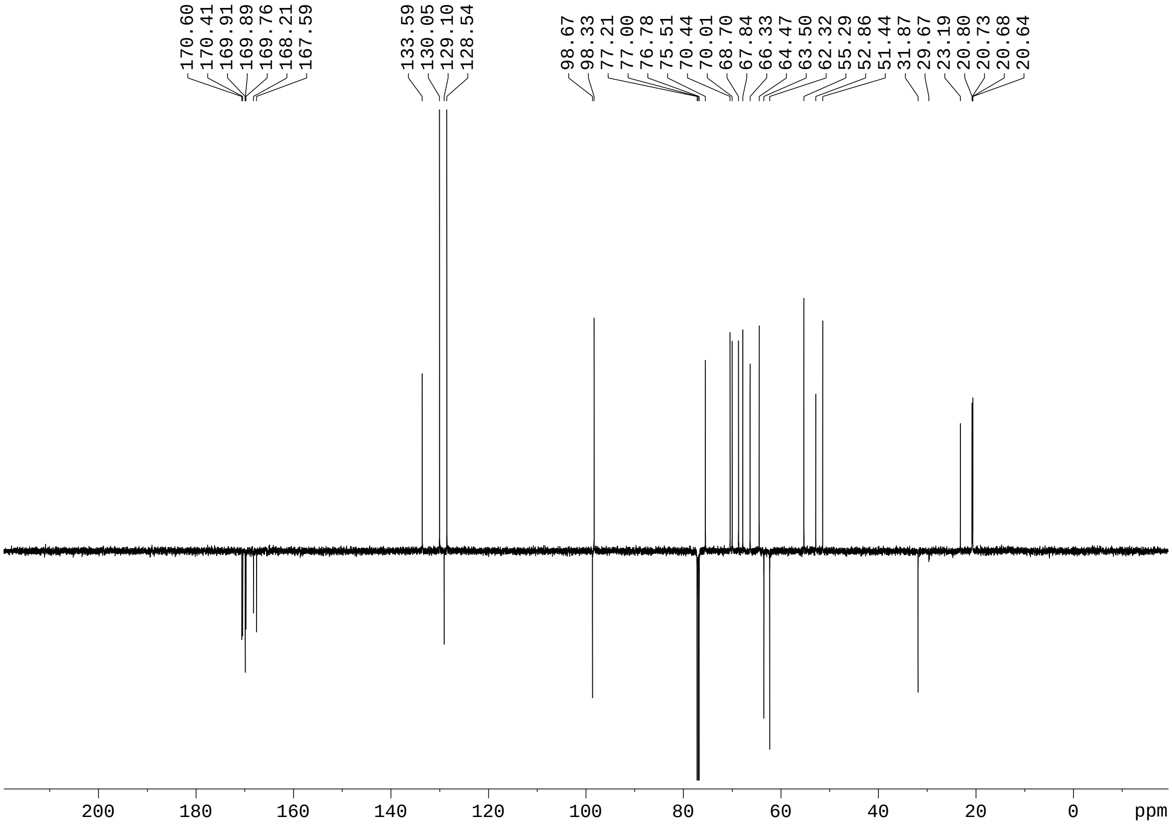

^{13}C NMR (CDCl$_3$, 150 MHz)

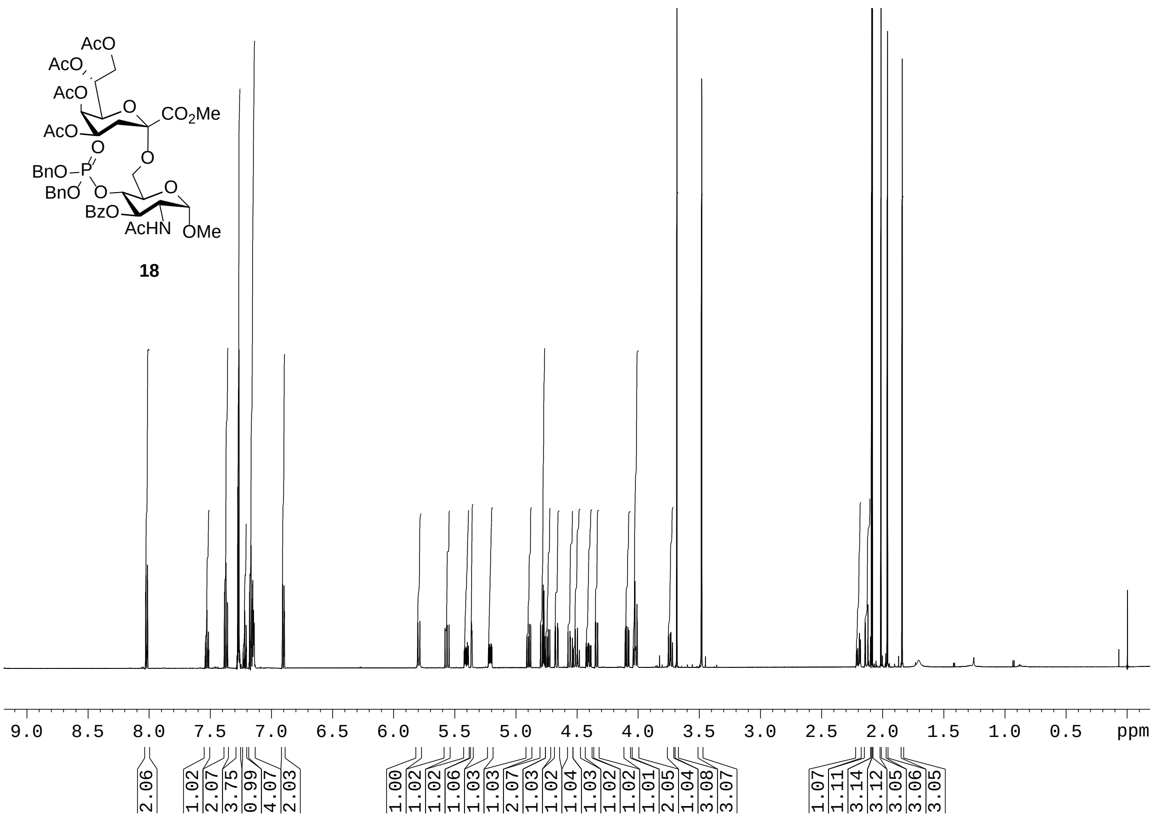

^{1}H NMR ($CDCl_3$, 600 MHz)

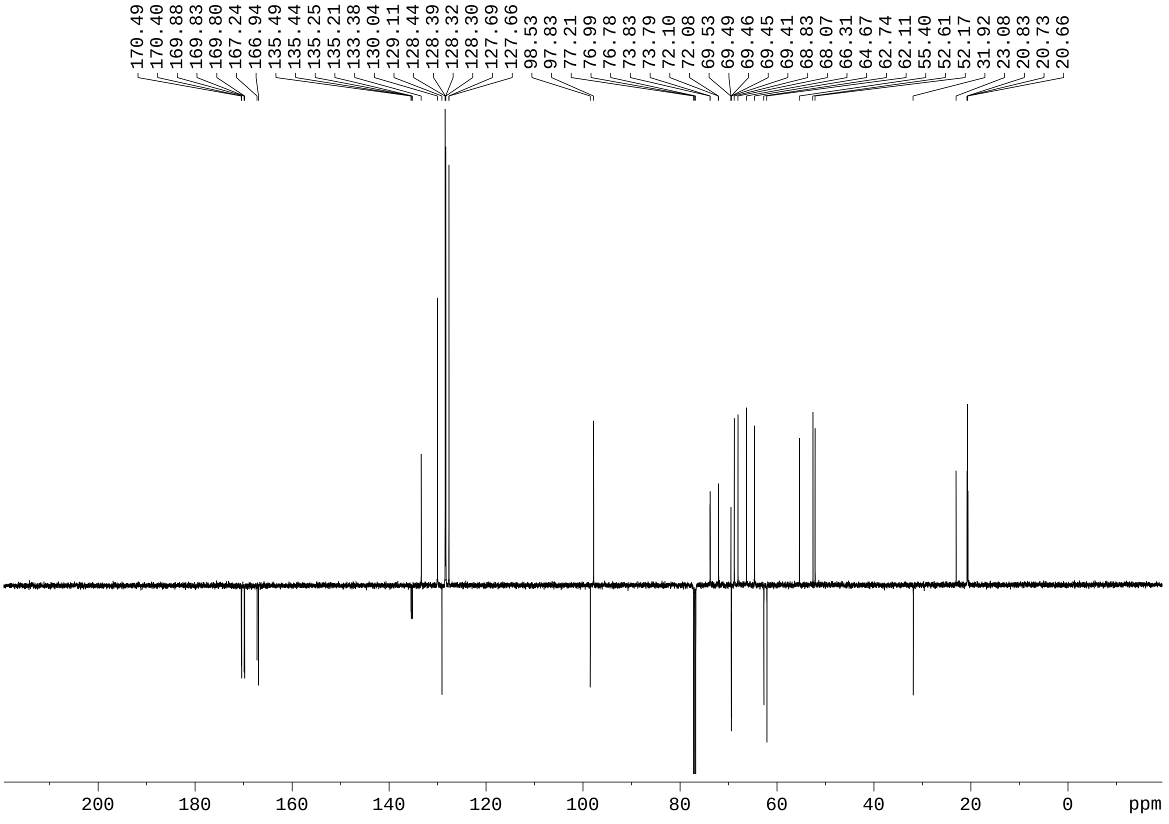

^{13}C NMR ($CDCl_3$, 150 MHz)

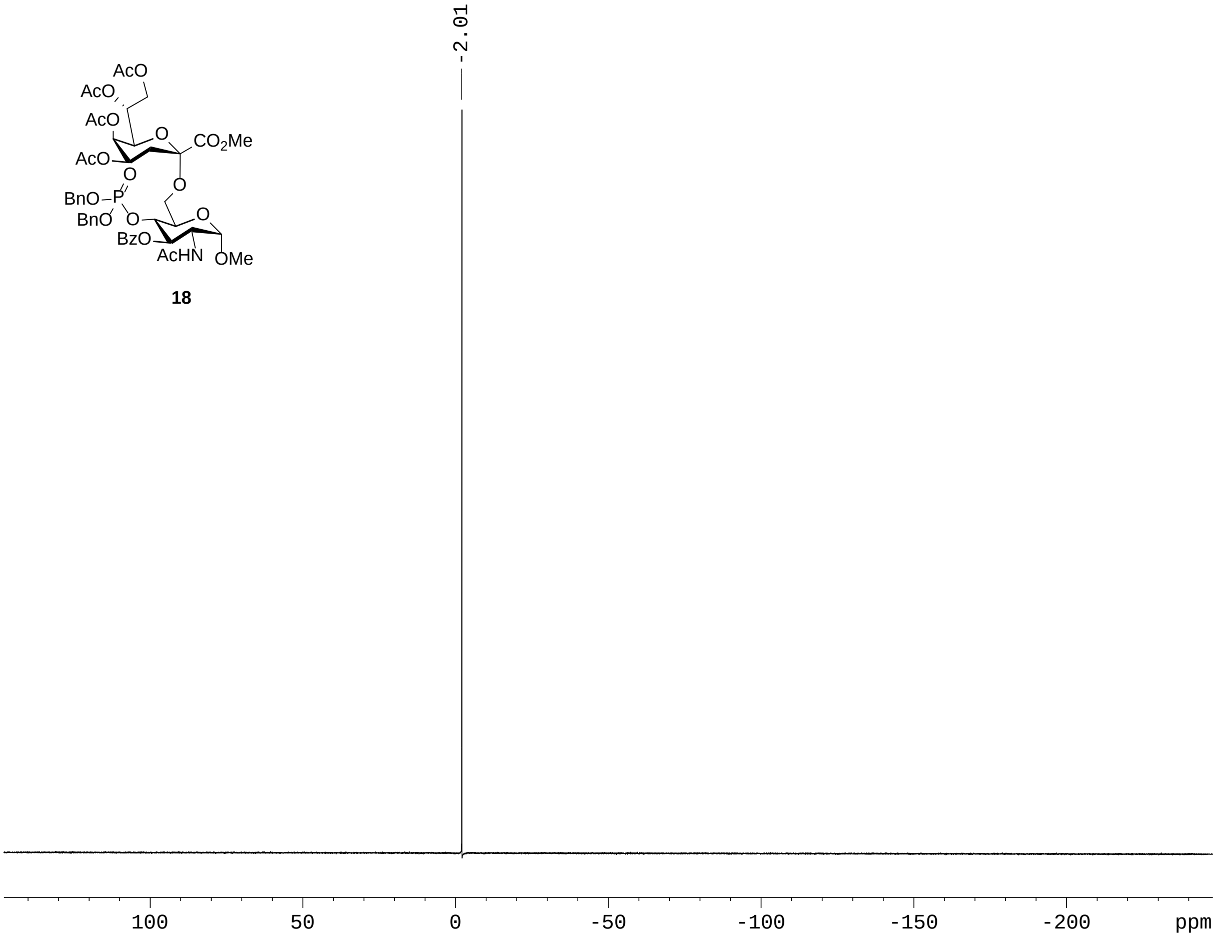

^{31}P NMR ($CDCl_3$, 243 MHz)

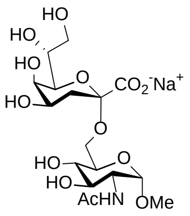

19

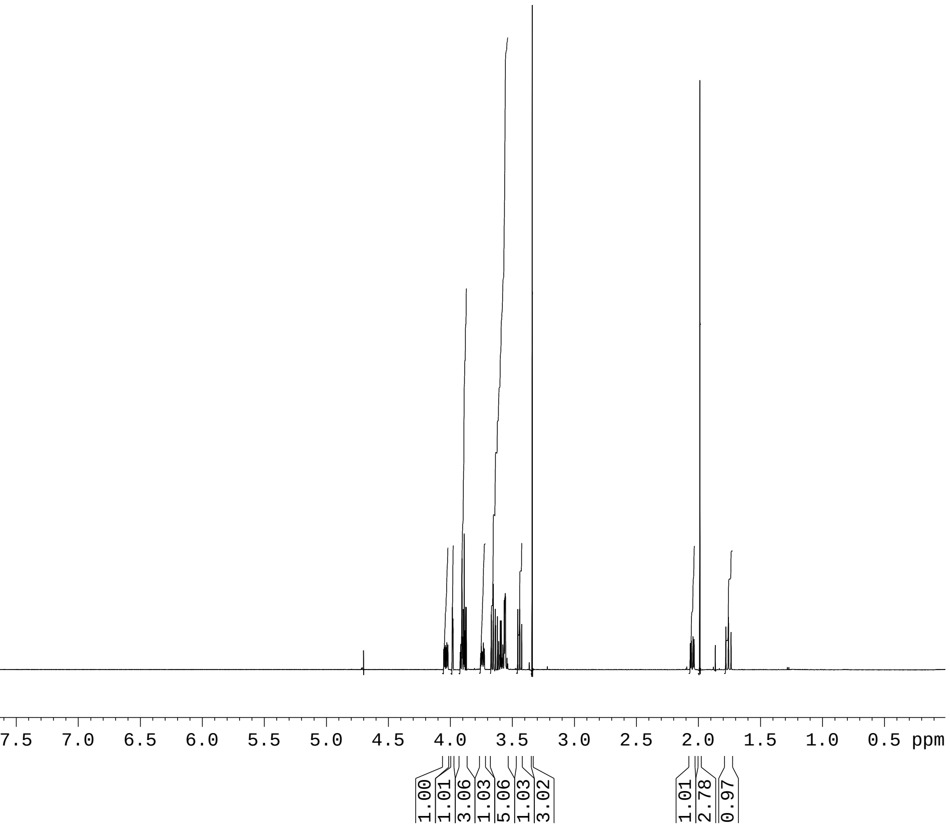

^{1}H NMR (D_2O, 600 MHz, pH ~ 7.0)

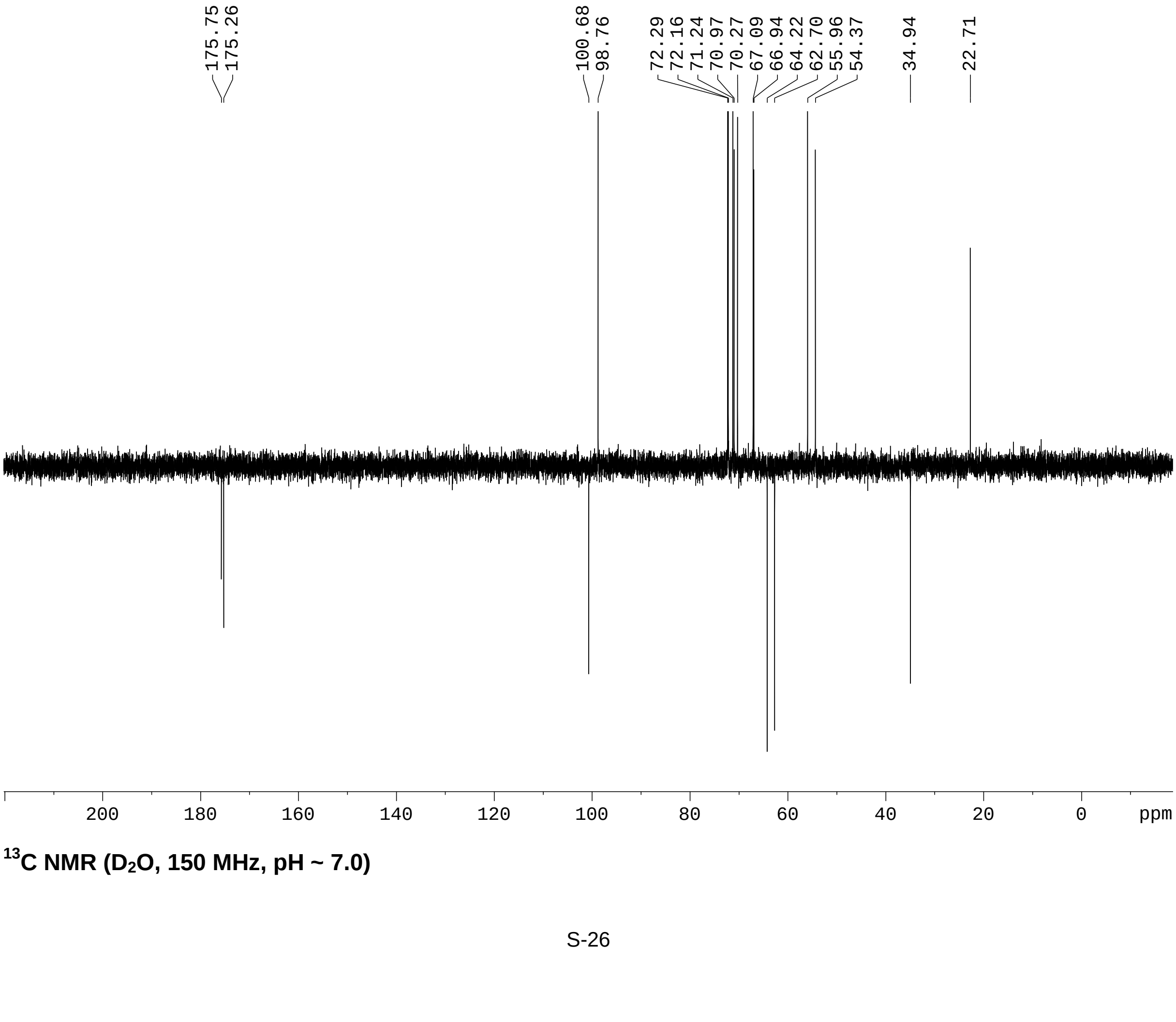

^{13}C NMR (D_2O, 150 MHz, pH ~ 7.0)

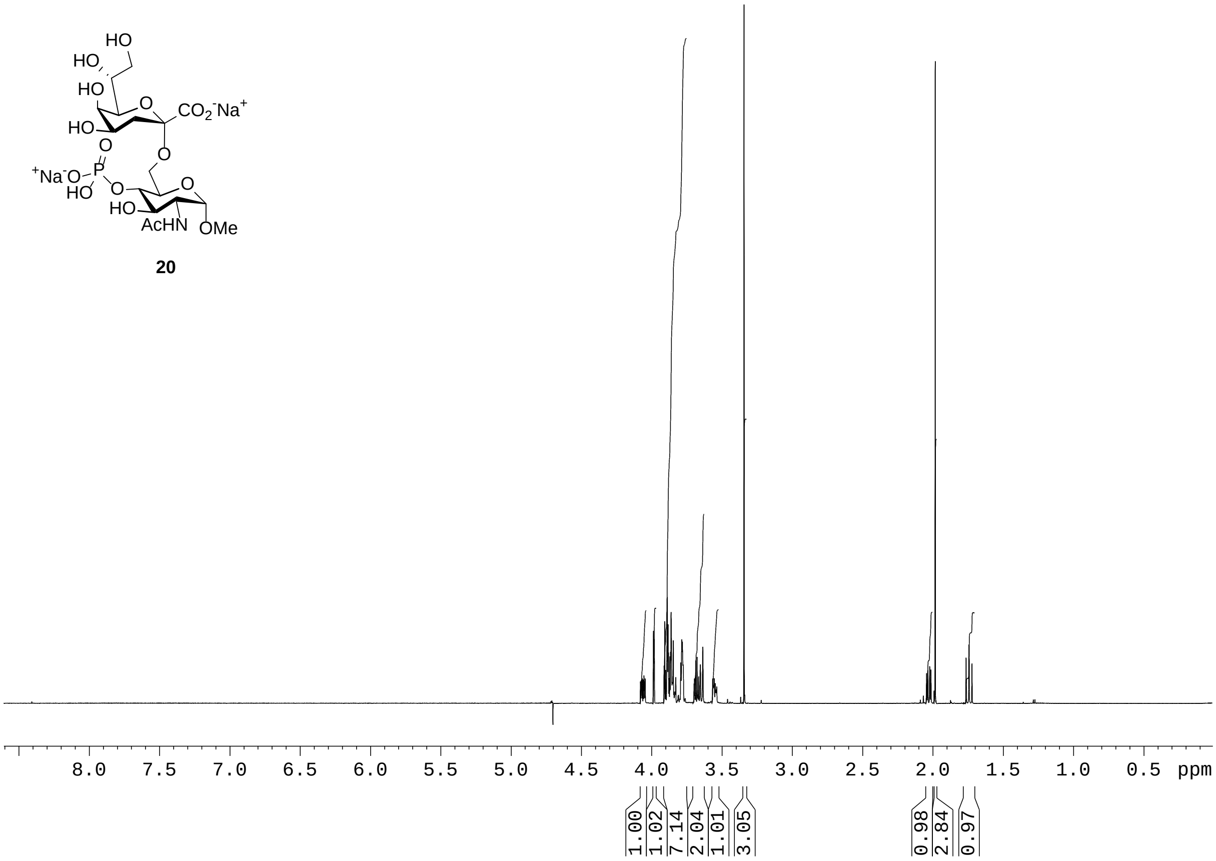

^{1}H NMR (D_2O, 600 MHz, pH ~ 7.0)

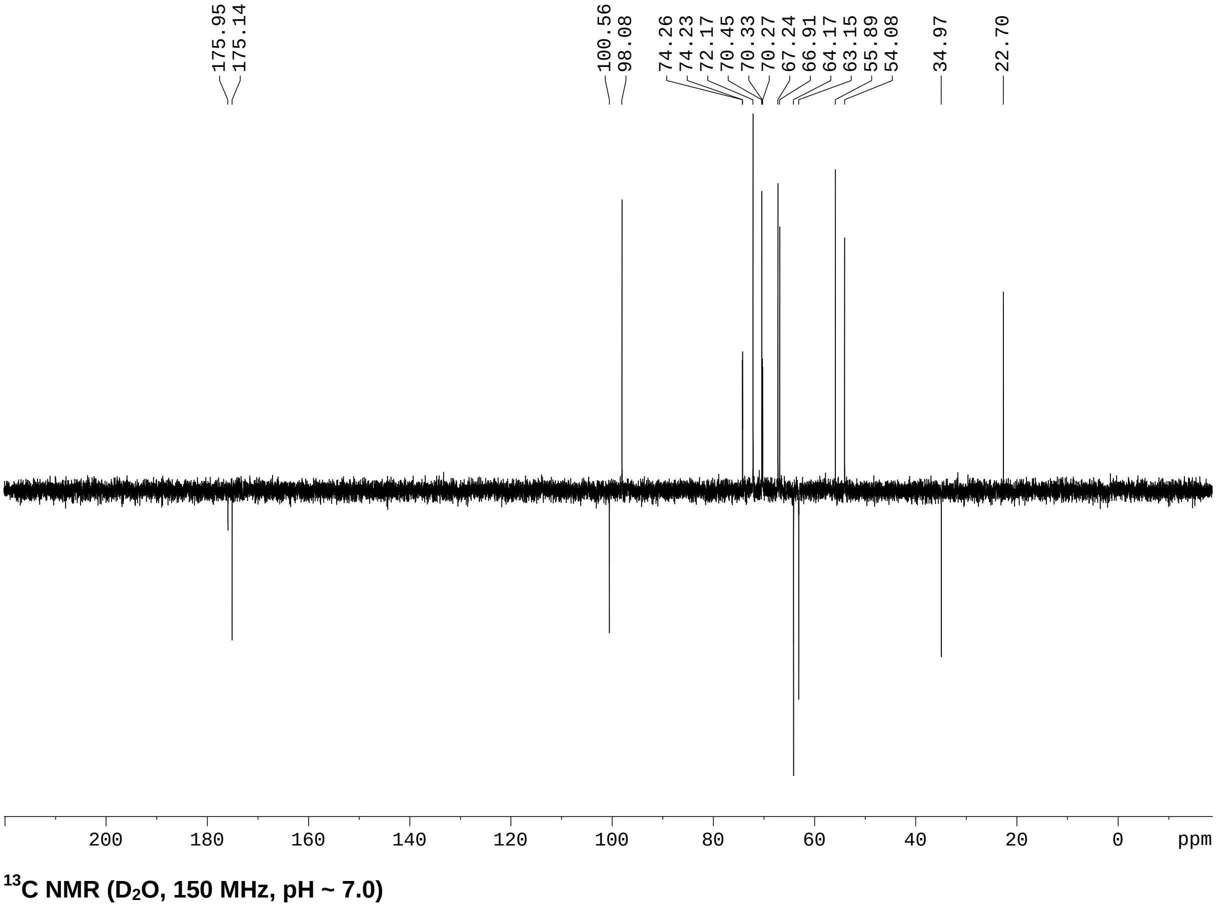

^{13}C NMR (D_2O, 150 MHz, pH ~ 7.0)

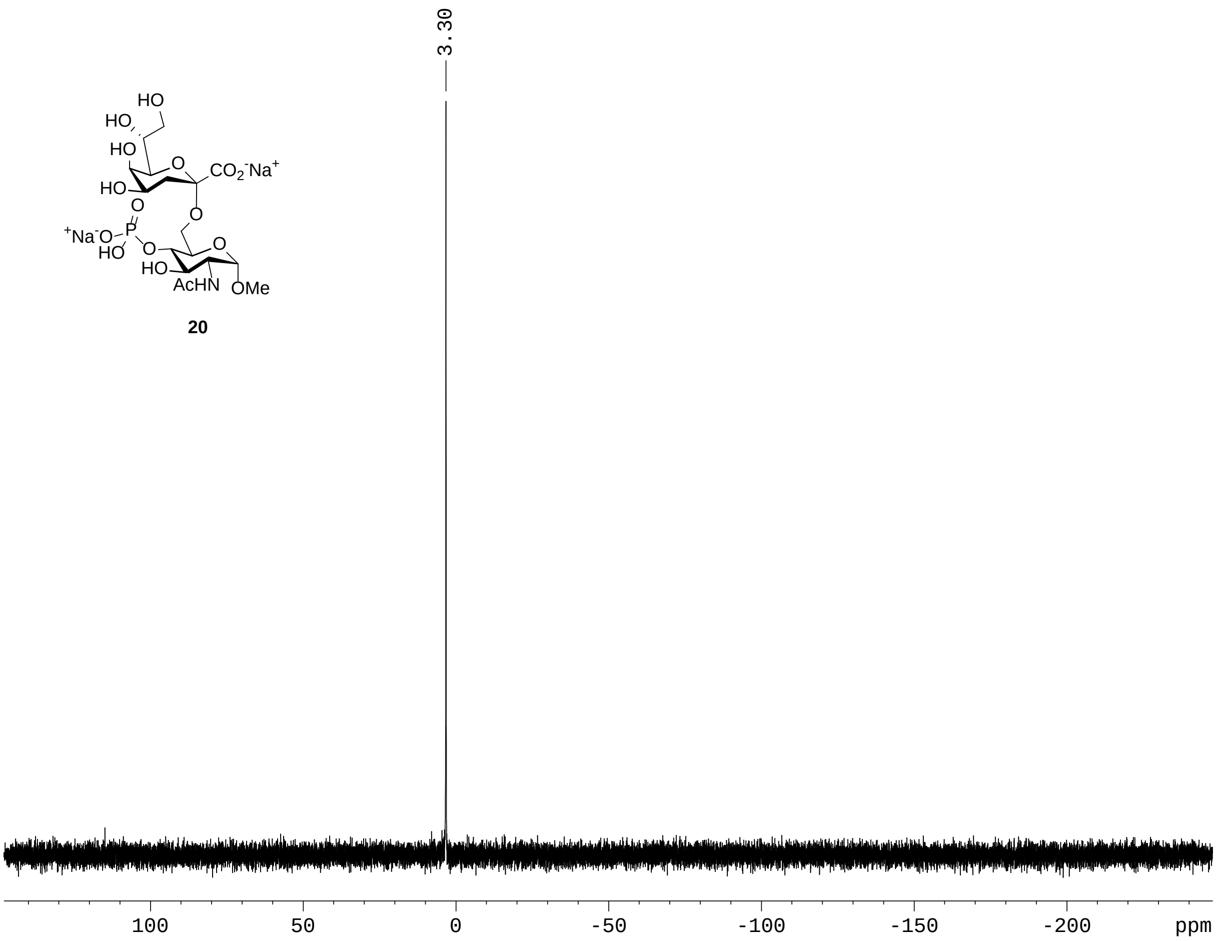

^{31}P NMR (D_2O, 243 MHz, pH ~ 7.0)

6. References

[S1] a) Y. Cai, C.-C. Ling, D. R. Bundle, *Org. Lett.* **2005**, *7*, 4021-4024; b) Y. Cai, C.-C. Ling, D. R. Bundle, *J. Org. Chem*. **2009**, *74*, 580-589.

[S2] P. Rollin, P. Sinay, *J. Chem. Soc. Perkin Trans. 1* **1977**, 2513 – 2517.

[S3] S. S. Rana, J. J. Barlow, K. L. Matta, *Carbohydr. Res*. **1983**, *113*, 257-271.

[S4] A. Scaffidi, K. A. Stubbs, R. J. Dennis, E. J. Taylor, G. J. Davies, D. J. Vocadlo, R. V. Stick, Org. Biomol. Chem. 2007, 5, 3013-3019.

[S5] R. W. Jeanloz, *J. Am. Chem. Soc*. **1954**, *76*, 555-558.

[S6] For the procedure with a similar compound see: T. Hadin, J. M. Pfeffer, A. J. Clarke, M. E. Tanner, *J. Org. Chem*. **2011**, *76*, 1118-1125.

[S7] L.-X. Wang, N. Sakairi, H. Kuzuhara, *Carbohydr. Res*. **1995**, *275*, 33-47.

[S8] M. B. Pinto, K. B. Reimer, D. G. Morissette, D. R. Bundle, *J. Org. Chem.* **1989**, *54*, 2650-2656.

[S9] Jpn. Kokai Tokkyo Koho JP 60 51, 702 [85 51, 702]; Chem. Abstr. 104, 34298j (**1985**)

[S10] T. Ogawa, T. Kitajima, T. Nukada, *Carbohydr. Res*. **1983**, *123*, C5-C7.

[S11] LC-MS data were obtained on a Shimadzu LC-MS-2020 quadrupole (ESI source) instrument.
